# Antibody response in snakes with boid inclusion body disease

**DOI:** 10.1371/journal.pone.0221863

**Published:** 2019-09-09

**Authors:** Katharina Windbichler, Eleni Michalopoulou, Pia Palamides, Theresa Pesch, Christine Jelinek, Olli Vapalahti, Anja Kipar, Udo Hetzel, Jussi Hepojoki

**Affiliations:** 1 Institute of Veterinary Pathology, Vetsuisse Faculty, University of Zurich, Zurich, Switzerland; 2 Department of Veterinary Pathology and Public Health, Institute of Veterinary Science, University of Liverpool, Liverpool, United Kingdom; 3 University of Helsinki, Faculty of Veterinary Medicine, Department of Veterinary Biosciences, Helsinki, Finland; 4 University of Helsinki, Faculty of Medicine, Medicum, Department of Virology, Helsinki, Finland; The Scripps Research Institute, UNITED STATES

## Abstract

Boid Inclusion Body Disease (BIBD) is a potentially fatal disease reported in captive boid snakes worldwide that is caused by reptarenavirus infection. Although the detection of intracytoplasmic inclusion bodies (IB) in blood cells serves as the gold standard for the *ante mortem* diagnosis of BIBD, the mechanisms underlying IB formation and the pathogenesis of BIBD are unknown. Knowledge on the reptile immune system is sparse compared to the mammalian counterpart, and in particular the response towards reptarenavirus infection is practically unknown. Herein, we investigated a breeding collection of 70 *Boa constrictor* snakes for BIBD, reptarenavirus viraemia, anti-reptarenavirus IgM and IgY antibodies, and population parameters. Using NGS and RT-PCR on pooled blood samples of snakes with and without BIBD, we could identify three different reptarenavirus S segments in the collection. The examination of individual samples by RT-PCR indicated that the presence of University of Giessen virus (UGV)-like S segment strongly correlates with IB formation. We could also demonstrate a negative correlation between BIBD and the presence of anti-UGV NP IgY antibodies. Further evidence of an association between antibody response and BIBD is the finding that the level of anti-reptarenavirus antibodies measured by ELISA was lower in snakes with BIBD. Furthermore, female snakes had a significantly lower body weight when they had BIBD. Taken together our findings suggest that the detection of the UGV-/S6-like S segment and the presence of anti-reptarenavirus IgY antibodies might serve as a prognostic tool for predicting the development of BIBD.

## Introduction

Boid inclusion body disease (BIBD) is a widespread disease of captive boid snakes known since the 1970s [1–3]. The disease is characterised by the presence of eosinophilic and electron-dense intracytoplasmic inclusion bodies (IBs) in most cell types of affected snakes [1–3]. In the early 2010s, we and others identified arenaviruses as the most likely causative agents of BIBD, by demonstrating that the IBs consist mainly of arenavirus nucleoprotein [4–7]. The causative link was later confirmed by experimental infection of boas and pythons with reptarenavirus isolates [8]. The family *Arenaviridae* in the order *Bunyavirales* currently comprises four genera: *Mammarenavirus*, *Reptarenavirus*, *Hartmanivirus*, and *Antennavirus* [9]. The arenaviruses found in snakes with BIBD belong to the genera *Reptarenavirus* and *Hartmanivirus* [9].

The genome of reptarenaviruses is a bi-segmented single-stranded negative-sense RNA with ambisense coding strategy. The small (S) segment encodes the nucleoprotein (NP) and the glycoprotein precursor (GPC), while the matrix protein (ZP) and the RNA-dependent RNA polymerase (RdRp) are encoded by the large (L) segment [10]. The genome of hartmaniviruses is similar, except that it lacks the ZP [10]. Snakes with BIBD are commonly co-infected with several reptarenaviruses, and, curiously, they often harbour more L than S segments [1,11,12]. The co-existence of multiple segments in an infected snake likely allows re-assortment of L and S segments [12]. The genetic variation between the known reptarenaviruses is tremendous and up to now L segments of approximately 30 different reptarenavirus species are known [1,10–12]. The genetic dissimilarity significantly hampers the development of sensitive “pan-reptarenavirus” RT-PCR tools. Therefore, since the IBs occur in blood cells including erythrocytes, IB detection in blood smears represents the current gold standard for *ante mortem* BIBD diagnosis [3,13]. However, the presence of IBs does not associate with pathological changes or clinical signs, and thus snakes with reptarenavirus infection can remain clinically healthy for a long time [4,8]. Subclinical infections together with horizontal and vertical transmission of reptarenaviruses [1,12] are the likely reasons behind reptarenavirus co-infections being rather a rule than an exception in snakes with BIBD.

Despite the above facts, BIBD appears to be ultimately lethal [1–3]. Clinical features observed in snakes with BIBD include neurological signs, regurgitation, anorexia, pneumonia, stomatitis, and lymphoproliferative disorders [2,13,14]. The pathogenesis is poorly understood, however, the fact that bacterial infections and/or neoplastic processes are common in snakes with BIBD suggests that the disease is associated with immunosuppression [2–4]. Lymphocytic choriomeningitis virus (LCMV), the prototype arenavirus (genus *Mammarenavirus*), induces immunosuppression by inhibition of type I interferon (IFN-I) production [15–17]. The underlying mechanism is prevention of the RIG-I(retinoic acid inducible gene-I)/MAVS(mitochondrial antiviral signaling) pathway by the NP of LCMV [10,17]. The IFN-I production is further inhibited by the ZP of LCMV, which enters the nucleus and induces re-localisation of promyelocytic leukemia (PML) bodies to the cytoplasm [10,18,19]. Intriguingly, PML bodies contribute to tumour suppression which is hampered by their cytoplasmic localisation [20], thus the ZP of reptarenaviruses could promote tumourigenesis by such a mechanism. Additionally, the ZP of New World arenaviruses prevents the type I IFN response by binding to RIG-I [17].

Currently, not much is known about the immune response of snakes to reptarenaviruses. In fact, the knowledge of the reptile immune response in general is scarce, mainly relying on individual studies undertaken on different species [21]. It has been shown that like all vertebrates, reptiles mount an innate and adaptive immune response, comprising both humoral and cell-mediated factors [21,22]. Like in mammals, the humoral branch of the reptile innate immune system relies heavily on antimicrobial peptides and proteins as well as the complement pathway [21]. Reptiles have equivalents of interleukins (IL), IFNs and Toll-like receptors and can therefore coordinate their immune response, however, *in vitro* studies show the reptile system to be temperature and hormone dependent [21,23–28]. Also, in contrast to mammals with their cytokine-mediated development of fever, snakes are poikilotherm and thus increase their body temperature behaviourally by exposing themselves to higher environmental temperatures as demonstrated by stimulation with bacterial LPS or infection with gram-negative bacteria [21,29,30].

The adaptive immune response of both mammals and reptiles has a cell-mediated and a humoral component. The former is based on T cells, and in reptiles their proliferation depends on the seasonal cycle [31–33]. Females show a stronger cell-mediated immunity than males in both mammals and reptiles [21,34–36], and in the latter T cell proliferation is stronger in non-gravid than in gravid animals [21,36]. In vertebrates, including reptiles, the immunoglobulins (Ig) orchestrate the humoral branch of the adaptive immune system. Reptiles produce Igs of three classes, IgY, IgM and IgD; the leopard gecko (*Eublepharis macularius*), for example, also produces IgA [21,37]. The reptile IgM is considered as equivalent to IgM of other vertebrates, and IgY corresponds to mammalian IgG [22,38]; the molecular features are similar. Depending on the snake species IgY may occur in three isotypes, a, b, and c. According to sequence analysis, the IgY isotypes of boid snakes differ from those of other snake species but show structural similarity to mammalian IgG in that the heavy and light chains are covalently bound [37]. In both reptiles and mammals exposure to an infectious agent (or other foreign antigen) triggers IgM production approximately within a week [21]. In mammals IgM appears around 10 days [21] and peaks around 10–14 days post exposure. In reptiles, serum IgM levels reach the peak much later, up to 8 weeks post exposure, indicating differences in the maturation of the adaptive immune response compared to mammals [14,21]. Depending on the species studied and the antigens used, the IgM response in reptiles can last up to 34 weeks after exposure [21], whereas the IgY response appears around 31 days post exposure and can last for many years, similar to the mammalian IgG response [39].

Overall, in comparison to mammals, the reptile antibody response is weaker [22] since the titres do not necessarily increase after a second antigen exposure and there is a lack of affinity maturation [21,22]. However, studies on colubrid snakes indicated an increase in titres after repeated antigen exposure [40], and the rapidness of the response indicates immunological memory [21,22,40]. Again, the reptile antibody response is affected by environmental and individual factors such as temperature, season, sex, age, and the neuroendocrine status [14,22].

We set up this study to assess the antibody response against reptarenaviruses in snakes. Our working hypothesis was that snakes with BIBD, i.e. with the presence of IBs in blood cells and confirmed reptarenvirus infection, would show low anti-reptarenavirus antibody titres, if any. We also wanted to study whether other measurable parameters, such as the sex, age, and weight of the animals, or the number of reptarenaviruses infecting an individual snake could be associated with IB formation. To answer these questions, we studied a cohort (N = 70) of snakes in a single breeding collection with previously confirmed BIBD cases.

## Results

### Diagnosis of BIBD based on the cytological examination of blood smears

**Fig 1:**
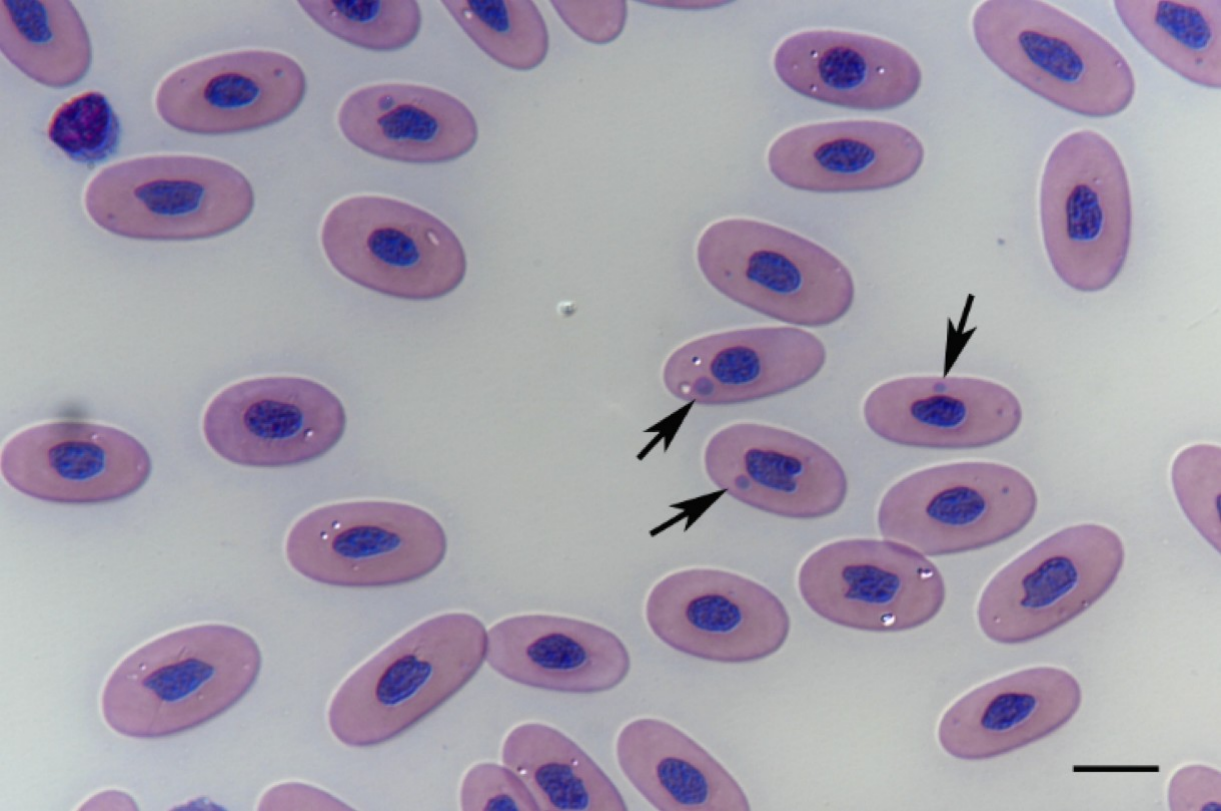
May-Grünwald-Giemsa stained blood smear, BIBD-positive snake (animal no. 1.25). Erythrocytes frequently exhibit intracytoplasmic inclusion bodies (arrows).

We based the BIBD diagnosis on the detection of IBs in cells in blood smears stained with May-Grünwald-Giemsa [7]. A similar approach was recently confirmed to correlate well with immunological staining of peripheral white blood cells (PWBC) for reptarenavirus NP [41]. We confirmed the association of the IBs with reptarenavirus infection by RT-PCR (see below), considering this as further proof of the disease and evidence that affected animals will eventually develop clinical signs [13]. We could detect IBs (Fig 1) in 34 of the 70 blood smears studied (48.57%; BIBD-positive snakes; Table 1). In the remaining 36 snakes (51.43%) the blood cells were free of IBs (BIBD-negative snakes; Table 2) [2]. At the time of blood sampling, all but the two debilitated snakes and the animal with cloacal prolapse (animals 1.18, 1.20, 1.29) appeared clinically healthy.

**Table 1:** Animals with BIBD included into the study (diagnosis based on the detection of intracytoplasmic inclusion bodies in blood cells, using blood smears).

Animal (number)	Age (years)	Sex	Weight (kg)	S segment			WB		ELISA					
							UGV-1		UGV-1		UHV NP		UHV NP-C	
				UGV-2	S5-like	TSMV-2	IgY	IgM	IgY	IgM	IgY	IgM	IgY	IgM
1.01	2	M	1.10	+	+	+	-	-	-	+	-	-	-	-
1.02	2	M	2.10	+	+	+	++	++	-	+	-	-	-	-
1.03	3	M	1.40	+	+	+	+	++	-	-	-	-	-	-
1.04	3	M	1.80	+	+	-	++	+	+	+	-	-	-	-
1.05	3	M	3.00	+	+	-	-	+	-	-	+	-	+	-
1.06	3	F	1.00	+	+	-	++	+	-	-	-	-	-	-
1.07	3	F	1.50	+	-	-	+++	+++	+	+	+	+	+	+
1.08	4	M	1.40	+	+	+	+	+	-	+	-	-	-	-
1.09	4	M	1.60	+	+	+	+++	-	-	+	-	-	-	-
1.10	4	M	2.20	+	+	+	++	+++	-	-	-	-	-	-
1.11	4	F	2.50	+	+	-	-	-	-	-	-	-	-	-
1.12	4	F	2.60	+	+	-	-	-	-	-	-	-	-	-
1.13	4	F	3.30	+	+	+	+	+	-	-	-	-	-	-
1.14	4	F	3.40	+	+	-	+	+	-	-	+	+	+	+
1.15	4	F	3.40	+	+	-	+	-	-	+	-	-	-	-
1.16	4	F	3.40	+	+	+	+	+++	-	+	-	-	-	-
1.17	4	F	3.70	+	+	+	-	-	-	-	-	-	-	-
1.18	4	F	3.90	+	+	+	+++	+++	+	+	+	+	+	+
1.19	4	F	4.10	+	+	-	+	+	-	-	-	-	-	-
1.20	5	M	0.90	+	+	+	-	++	-	+	-	+	-	+
1.21	5	M	1.10	+	+	+	-	-	-	+	-	-	-	-
1.22	5	M	1.60	+	+	+	+++	+++	+	+	-	-	+	+
1.23	5	M	2.80	+	-	-	-	++	-	+	-	-	-	-
1.24	5	F	1.70	+	+	+	+	++	-	-	-	-	-	-
1.25	5	F	2.60	+	+	+	-	+	-	-	-	-	-	-
1.26	5	F	4.50	+	+	+	+	++	-	-	-	-	-	-
1.27	6	M	1.80	+	+	+	+	+	-	+	-	-	+	-
1.28	6	M	3.20	+	+	+	-	++	+	+	-	-	-	+
1.29	6	F	2.70	+	+	+	++	-	-	+	-	+	-	+
1.30	6	F	5.50	+	+	+	+++	+++	+	+	-	+	-	+
1.31	7	F	9.00	+	+	+	+	+	+	n.a.	-	-	-	-
1.32	n.a.	M	2.40	+	+	+	+	+	-	+	+	-	-	+
1.33	n.a.	M	2.70	-	+	+	+	-	+	+	+	+	+	+
1.34	n.a.	M	3.10	+	+	+	++	++	+	-	-	-	-	-

n.a.–not available; F–female; M–male; S segment–reptareavirus S segment determined by RT-PCR; WB–Western Blot; Western Blot results graded according to signal intensity:—(negative), + (weakly positive), ++ (moderately positive), +++ (strongly positive); ELISA–Enzyme linked immunosorbent assay.

**Table 2:** Animals without BIBD included into the study (diagnosis based on the detection of intracytoplasmic inclusion bodies in blood cells, using blood smears).

Animal (number)	Age (years)	Sex	Weight (kg)	S segment			WB		ELISA					
							UGV-1		UGV-1		UHV NP		UHV NP-C	
				UGV-2	S5-like	TSMV-2	IgY	IgM	IgY	IgM	IgY	IgM	IgY	IgM
2.01	2	M	0.9	-	+	+	++	+	-	+	+	+	+	+
2.02	2	M	1.5	-	+	+	++	+++	-	+	+	-	+	+
2.03	2	F	1.3	-	+	+	-	+	-	-	-	-	+	+
2.04	3	M	1.2	-	+	+	+++	++	+	+	-	-	+	+
2.05	3	M	1.3	+	+	+	-	-	-	+	+	-	+	+
2.06	3	M	1.7	-	+	+	+	+++	-	-	-	-	-	-
2.07	3	M	1.8	-	+	+	-	++	-	+	-	-	+	-
2.08	3	F	2.2	-	+	+	-	-	-	+	-	-	-	-
2.09	4	M	2.1	+	+	+	+++	++	+	-	+	-	-	-
2.10	4	M	2.7	+	+	+	+++	+++	-	+	-	+	-	-
2.11	4	M	3.3	+	+	+	++	++	+	-	+	+	+	+
2.12	4	F	3.7	-	+	+	+++	+++	+	+	+	-	+	+
2.13	4	F	3.8	-	+	+	++	++	+	+	+	+	+	+
2.14	4	F	5.8	-	+	+	+++	++	+	+	-	-	-	-
2.15	4	F	6.8	-	-	-	-	-	-	-	-	-	-	-
2.16	5	M	2.2	+	+	+	-	-	+	-	+	-	+	+
2.17	5	M	2.5	-	+	+	+	+	-	-	+	+	+	+
2.18	5	F	5.0	+	-	+	-	+	+	-	-	-	-	-
2.19	5	F	5.3	-	+	-	++	+	+	+	-	-	-	-
2.20	5	F	5.3	-	-	-	+	-	+	-	-	-	-	-
2.21	5	F	5.5	-	+	+	++	-	+	-	+	-	+	+
2.22	5	F	5.7	-	-	+	++	-	+	+	+	+	+	+
2.23	5	F	6.1	-	+	+	+++	+	+	-	+	-	-	-
2.24	6	M	2.5	+	+	+	-	-	+	-	-	-	-	-
2.25	6	M	3.4	+	+	+	-	-	+	+	-	-	-	-
2.26	6	M	3.5	-	+	+	-	+	-	-	-	-	-	-
2.27	6	F	3.1	-	-	-	+++	+++	+	+	+	+	+	-
2.28	6	F	5.6	-	+	+	+++	+++	+	-	-	-	-	-
2.29	7	M	3.3	+	+	-	-	++	-	-	-	-	-	-
2.30	7	M	4.0	-	+	+	+++	+	+	-	-	-	-	-
2.31	7	F	5.0	+	-	+	+++	+++	+	-	+	-	+	-
2.32	7	F	7.0	-	+	+	+++	+++	+	-	+	+	+	+
2.33	7	F	7.5	-	+	+	++	++	+	+	-	-	-	+
2.34	7	F	10.0	-	+	+	+++	+++	+	-	+	+	+	+
2.35	8	M	3.4	-	+	+	+++	+	+	-	+	+	+	+
2.36	8	F	7.0	-	-	-	+++	++	+	+	-	-	+	+

n.a.–not available; F–female; M–male; S segment–reptareavirus S segment determined by RT-PCR; WB–Western Blot; Western Blot results graded according to signal intensity:—(negative), + (weakly positive), ++ (moderately positive), +++ (strongly positive); ELISA–Enzyme linked immunosorbent assay.

**Fig 2:**
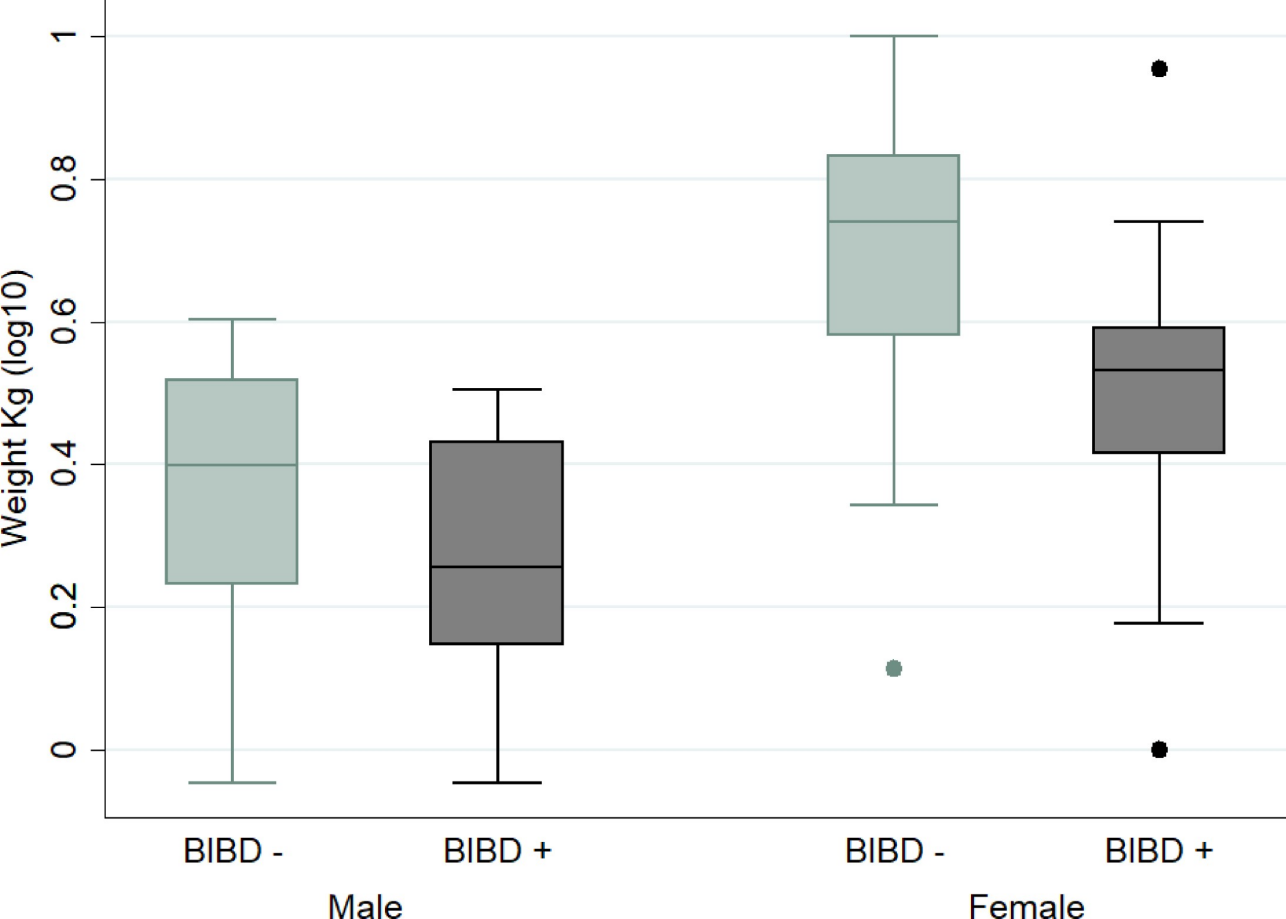
Association of BIBD, sex and body weight.

We examined the animals’ age and weight against the BIBD diagnosis (Table 3). The average age was 4.6 years (95%CI: 4.26–4.99). We did not find statistically significant differences in age between female and male animals or between BIBD-positive and -negative animals. However, we found a statistically significant (p<0.01) association between BIBD and the weight of the female animals: BIBD-positive female animals had significantly lower body weights (Fig 2); the geometric mean of the weight was 3.077kg for the BIBD-positive female animals and 4.912 kg for the negative ones. The same association was not significant for male animals (Table 3). Linear regression established that the weight of the animals was significantly associated with age, sex and BIBD status (Table 4), F(3,63) = 39.67, and they accounted for 63.74% of weight variability. The regression equation is: Predicted Weight = -0.177 + 0.084 age + 0.255 sex—0.107 BIBD-positive.

**Table 3:** Results of inclusion body detection in blood cells (i.e. diagnosis of BIBD) against population parameters. Univariate analysis and stratification by sex.

	Sex (Row%) (Col%)			Weight* (95% CI) N = 70			Age**(n) (95% CI) N = 67		
	M	F	All	M	F	All	M	F	All
**BIBD -**	**17** (47.22%) (50.00%)	**19** (52.78%) (52.78%)	**36** (100.00%) (51.43%)	**2.238** (1.788–2.801)	**4.912** (3.919–6.156)	**3.389** (2.767–4.149)	**4.588** (17) (3.643–5.534)	**5.211** (19) (4.464–5.957)	**4.917** (36) (4.343–5.491)
**BIBD +**	**17** (50.00%) (50.00%)	**17** (50.00%) (47.22%)	**34** (100.00%) (48.57%)	**1.876** (1.532–2.297)	**3.077** (2.373–3.991)	**2.403** (2.010–2.873)	**4.071** (14) (3.305–4.838)	**4.471** (17) (3.922–5.019)	**4.290** (31) (3.854–4.726)
**All**	**34** (48.57%) (100.00%)	**36** (51.36%) (100.00%)	**70** (100.00%) (100.00%)	**2.049** (1.770–2.372)	**3.938** (3.287–4.719)	**2.867** (2.497–3.293)	**4.355** (31) (3.759–4.950)	**4.861** (36) (4.395–5.327)	**4.627** (67) (4.260–4.994)
χ2 = 0.054, p = 0.816				t = 1.2365, df = 32 p = 0.2253	t = 2.8801, df = 34 **p<0.01**	t = 2.5748, df = 68 **p<0.05**	t = 0.8785, df = 29 p = 0.3869	t = 1.6494, df = 34 p = 0.1083	t = 1.7226, df = 65 p = 0.0897

*Kg, geometric mean

**Years

**Table 4:** Multiple linear regression: Factors associated with weight (Kg log10) (n = 67).

Factors	Adjusted *b* (95% CI)	*P*-value
Sex (Female)	0.255 (0.178–0.333)	< 0.001
Inclusion detection (positive)	-0.107 (-0.185 –- 0.293	< 0.01
Age (years)	0.084 (0.058–0.110)	< 0.001
Multiple linear regression (*AdjR*^2^ = 0.6374).		

### Characterization of the breeding collection’s “reptarenavirome”

We and others have previously reported that snakes with BIBD often harbour several reptarenavirus L and S segments; usually, more L than S segments are found in each snake [1,11,12]. To study whether the BIBD-negative snakes would also be free of reptarenavirus infection, we performed a meta-transcriptomic analysis of pooled blood samples (one pool from three snakes without evidence of IBs in blood cells, the other from three snakes with a high number of IBs in blood cells). From the reads acquired by NGS of the BIBD-positive blood pool we could assemble five reptarenavirus L segments and one S segment, as well as two pairs of hartmanivirus L and S segments [10]. To our surprise, we could not assemble any full-length L or S segments from the reads acquired from the BIBD-negative blood pool. However, using a mapping approach we identified some reads matching the L and S segments assembled from the data of the BIBD-positive blood pool. We then decided to screen a further three pools of three blood samples by RT-PCR, using virus-specific primers from our earlier study [1], one pool from BIBD-negative snakes, two from BIBD-positive snakes. We found the S segments of UGV-2, S5-like, and TSMV-2 to be present in the positive pools, while the negative pool was only positive for the latter two. The L segment profiles of the pools seemed variable.

We analysed the population parameters against the RT-PCR test results for associations with the detection of hartmaniviruses (OScV-1 and -2). OScV-1 detection did not significantly associate with any of the population parameters, while OScV-2 detection positively associated with age. The average age of animals without OScV-2 infection was 4.28 years (n = 50, 95%CI: 3.895–4.665), whereas it was 5.647 (n = 17, 95%CI: 40260–4.994) for OScV-2 positive snakes (t = -3.498, df: 65, p<0.05). None of the other population parameters showed any associations with OScV-2 after controlling for age. OScV-1 and -2 detection showed poor to slight agreement with the other tests (Cohen’s kappa < 0.2).

### Detection of reptarenavirus S segments in individual samples by RT-PCRs

Reptarenaviruses require both segments to make infectious particles; therefore, we applied specific RT-PCR for the above identified three S segments to all animals to recognise the reptarenavirus infected, viraemic snakes. Of the 70 animals tested, we found 66 (94.3%) to exhibit reptarenavirus viraemia. Thirty snakes (42.9%) carried all three S segments examined (UGV-/S6-like, S5-like, and TSMV-2), and 32 (45.7%) carried two S segments. Of these, 21 snakes (30%) showed a combination of the S5-like and TSMV-2 S segments, nine snakes (12.9%) had the UGV-/S6-like and S5-like S segments, and two snakes (2.9%) had the UGV-/S6-like and TSMV-2 S segments. Of the four snakes with a single S segment, we found the UGV-/S6-like S segment in two, and the S5-like and TSMV-2 S segment in one snake each. The results are presented in detail in Table 1 and are summarised in Table 5.

**Table 5:** Summary of RT-PCR results including test agreement and sensitivity/specificity with inclusion detection considered the gold standard.

		BIBD		Total	Cohen’s κ	Sensitivity (95%CI)	Specificity (95%CI)
		+ve	-ve				
UGV-2	+ve	33	10	43	κ = 0.688 (0.524–0.852)	97.06% (93.10–100)%	72.22% (61.73–82.71)%
	-ve	1	26	27			
S5-like	+ve	32	29	61	κ = 0.133 (-0.019–0.284)	94.12% (88.61–99.63)%	19.44% (10.17–28.72)%
	-ve	2	7	9			
TSMV-2	+ve	24	30	54	κ = -0.125 (-0.320–0.069)	70.59 (59.91–81.26)%	16.67% (7.94–25.40)%
	-ve	10	6	16			
Any segment	+ve	34	32	66	κ = 0.108 (0.005–0.212)	100%	11.11% (3.75–18.47)%
	-ve	0	4	4			
Total		34	36	70			

We confirmed reptarenavirus viraemia in all BIBD-positive animals, and the majority (23/34; 67.65%) of these snakes carried all three S segments examined (UGV-/S6-like, S5-like, TSMV-2). Nine BIBD-positive snakes (26.47%) carried two S segments, and we detected only the UGV-/S6-like S segment in the remaining two animals (5.88%; animals 1.07 and 1.23) (Tables 1 and 5). The UGV-like S segment was present in BIBD-positive animals.

In BIBD-negative snakes (N = 36), we found all three viral S segments in seven snakes (19.4%), whereas 23 animals (63.9%) carried two S segments, and two snakes (5.56%) had a single S segment, one had the S5-like (animal 2.19) and the other the TSMV-2 (animal 2.22) S segment. Four snakes (11.1%) were negative for each S segment and deemed to be reptarenavirus-free (Tables 1 and 5).

Substantial agreement was identified between BIBD+ status and UGV-/S6-like S segment RT-PCR results (Cohen’s κ = 0.6878). The agreement of the remaining RT-PCR tests with BIBD is slight (S5-like κ = 0.1327, TMSV-2 κ = 0.1254, any segment detection κ = 0.183, Table 5). Sensitivity and specificity calculations are included in Table 5, though the study was not designed for such calculations.

We examined the associations of the RT-PCR results with population parameters (Table 6). Female animals positive for the UGV-/S6-like S segment, as expected given the test agreement with the presence of IB, have a significantly lower body weight (t = 2.99624882, df = 34, p<0.05). For male animals the difference in weight is not significant. There is no significant difference in the age of UGV-/S6-like S segment RT-PCR-positive and -negative animals or in their sex distribution. Multiple linear regression established that the age, sex and a positive UGV-/S6-like S segment RT-PCR result are significantly associated with the weight of the animals, F (3,63) = 36.98, and they accounted for 62.06% of weight variability. The regression equation is: Predicted Weight = -0.287 + 0.089 age + 0.235 sex—0.086 UGV-/S6-like indicating that the weight of UGV-/S6-like positive animals is lower than the weight of negative snakes after controlling for age and sex.

**Table 6:** RT-PCR results against population parameters, univariate analysis including stratification by sex.

	Sex (Row%) (Col%)			Weight* (95% CI) N = 70			Age**(n) (95% CI) N = 67		
	**M**	**F**	**Total**	**M**	**F**	**Total**	**M**	**F**	**Total**
**UGV** **RT-PCR** **-ve**	**10** (37.04%) (29.41%)	**17** (62.96%) (47.22%)	**27** (100.00%) (38.57%)	**2.090** (1.466–2.981)	**4.901** (3.796–6.328)	**3.575** (2.772–4.609)	**4.333** (9) (2.615–6.052)	**5.118** (17) (4.307–5.928)	**4.846** (26) (4.108–5.583)
**UGV** **RT-PCR** **+ve**	**24** (55.81%) (70.59%)	**19** (44.19%) (52.78%)	**43** (100.00%) (61.43%)	2.032 (1.720–2.400)	3.238506 2.544208 4.122274	**2.497** (2.142–2.910)	**4.355** (22) (3.759–4.969)	**4.632** (19) (4.070–5.193)	**4.488** (41) (4.088–4.555)
	χ^2^ = 2.341, p = 0.126			t = 0.1768, df = 32 p = 0.8608	t = 2.4882, df = 34 **p<0.05**	t = 2.622, df = 68 **p<0.05**	t = -0.046, df = 29 p = 0.9633	t = 1.0597, df = 34 p = 0.2968	t = 0.9485, df = 65 p = 0.346
**S5-like** **RT-PCR** **-ve**	**1** (11.11%) (2.94%)	**8** (88.89%) (22.22%)	**9** (100.00%) (12.86%)	**2.800**	**4.497** (2.937–6.884)	**4.266** (2.900–6.275)	**5.000** (1)	**5.375** (8) (4.039–6.711)	**5.333** (9) (4.180–6.486)
**S5-like** **RT-PCR** **+ve**	33 (54.10%) (97.06%)	28 (45.90%) (77.78%)	61 (100.00% (87.14%)	**2.030** (1.748–2.357)	**3.792** (3.070–4.684)	**2.704** (2.336–3.130)	4.333 (30) (3.718–4.948)	4.714 (28) (4.210–5.219)	**4.517** (58) (4.126–4.909)
	χ^2^ = 5.8019, **p<0.05**			t = …, df = 32 p = …	t = 0.791, df = 34 p = 0.4344	t = 2.264, df = 68 **p<0.05**	t = …, df = 29 p = . . . .	t = 1.2051, df = 34 p = 0.2365	t = 1.528, df = 65 p = 0.131
**TSMV-2** **RT-PCR** **-ve**	**4** (25.00%) (11.76%)	**12** (75.00%) (33.33%)	**16** (100.00%) (22.86%)	**2.658** (1.734–4.074)	**3.338** (2.301–4.842)	**3.153** (2.383–4.172)	**4.500** (4) (1.453–7.547)	**4.500** (12) (3.622–5.378)	**4.5** (16) (3.722–5.278)
**TSMV-2** **RT-PCR** **+ve**	30 (55.56%) (88.24%)	24 (44.44%) (66.67%)	54 (100.00%) (77.14%)	**1.979** (1.688–2.321)	**4.278** (3.464–5.283)	**2.788** (2.368–3.282)	**4.333** (27) (3.694–4.973)	**5.042** (24) (4.395–5.619)	**4.667** (51) (4.236–5.098)
	χ^2^ = 4.6133, **p<0.05**			t = 1.3371, df = 32 p = 0.1906	t = -1.3264, df = 34 p = 0.1936	t = 0.743, df = 68 p = 0.460	t = 1.1885, df = 29 P = 0.8518	t = -1.1170, df = 34 p = 0.2718	t = -0.384, df = 65 p = 0.702
**Total**	**34** (48.57%) (100.00%)	**36** (51.43%) (100.00%)	**70** (100.00%) (100.00%)	**2.049** (1.770–2.372)	**3.938** (3.287–719)	**2.867** (2.497–3.293)	**4.355** (31) (3.759–4.950)	**4.861** (36) (4.395–5.329)	**4.626** (67) (4.260–4.994)

*Kg, geometric mean

**Mean Years

There is no significant difference in the age of S5-like S segment RT-PCR-positive and -negative animals but there are significantly more male positive animals (χ^2^ = 5.8019, p<0.05). The animals’ weight is not significantly associated with a positive S5-like S segment RT-PCR result after controlling for sex and age. There is no significant difference in the age of TMSV-2 S segment RT-PCR-positive and -negative animals. There are though significantly more male animals positive for the TMSV-2 S segment (χ^2^ = 4.435, p<0.05). The animals’ weight is not significantly associated with a positive TMSV-2 S segment RT-PCR result after controlling for sex and age.

Univariate analysis indicated that the number of S segments detected is not significantly associated with the age of the animals (ANOVA: F(6,66) = 1.17, p = 0.333). Male animals had significantly more S segments (mean = 2.559 [95%CI: 2.236–2.755]) than female animals (mean = 1.972 [95%CI:1.664–2.280]), (p<0.01). Linear regression indicates that the number of segments is negatively associated with the weight of the animals (F(1,68) = 8.83, R^2^ = 0.103, Predicted weight = 0.696–0.106 number of segments, p<0.01). When the confounding effect of sex was examined by stratifying for sex, no significant association was identified between the number of S segments and the animals’ weight. There is a positive association between the number of segments and the detection of IB in blood cells. The mean number of segments for BIBD-positive animals is 2.618 (95%CI: 2.407–2.828) and for BIBD-negative animals 1.917 (95%CI: 1.632–2.201) (p<0.001).

### Antibody response against reptarenavirus NP

So far, not much is known about the antibody response against reptarenaviruses in snakes. In our first report on identification of reptarenaviruses in snakes with BIBD, we used an indirect ELISA to indicate that there might be antibodies in some snakes with BIBD [7]. In a more recent study, we generated tools for the detection of IgM and IgY class antibodies in boas, and, using immunofluorescence and western blot, demonstrated that some BIBD-positive snakes have antibodies against reptarenavirus NP [14].

#### Antibody detection by western blot (WB)

We studied the plasma samples of the entire collection using WB as the detection tool, and used concentrated UGV-1 virions as the antigen. The main protein component of the virions is NP, which is why we interpret the signals as anti-NP IgY and IgM. The signal intensities varied and we applied the following grading: negative (–), weakly positive (+), moderately positive (++), and strongly positive (+++); the WB result for each snake is included in Table 1. Among the 34 BIBD-positive snakes, we found five (14.7%) negative for both anti-NP IgY and IgM, whereas 20 snakes (58.8%) had both anti-NP IgM and IgY antibodies, and nine (26.5%) had either anti-NP IgY (N = 4) or IgM (N = 5). Ten snakes were anti-NP IgY-negative and nine were anti-NP IgM-negative. The 36 BIBD-negative snakes included 22 (61.1%) anti-NP IgY- and IgM-positive snakes, eight (22.2%) were positive for either anti-NP IgY (N = 3) or IgM (N = 5), six (16.7%) were negative for both. Eleven snakes were anti-NP IgY-negative and nine anti-NP IgM negative. Within the entire collection 11 snakes were negative for both anti-NP IgY and IgM antibodies. There are no significant associations of WB results for NP IgY or IgM and any of the population parameters.

The WB results for anti-NP IgY and IgM in relation to BIBD are summarised in Table 7. The agreement of the WB results with BIBD is slight for anti-NP IgY (Cohen’s κ = 0.0294) and poor for IgM (κ = 0.0000). As for the RT-PCR results we included indicative sensitivity and specificity calculations. The sensitivity of the IgY WB in detecting BIBD is 70.6% (95%CI: 59.8%– 81.4%) and the specificity 32.4% (95%CI:21.2%– 64.3%). For IgM, the WB sensitivity is 73.5% (95%CI:63.0% - 84.0%) and the specificity 26.5% (95%CI:16.0% - 37.0%). We examined the agreement of the BIBD status against the graded WB results using Cohen’s weighted kappa(κ(w). For anti-NP IgY κ(w) is 0.0119 and for IgM κ(w) is 0.000 indicating slight and poor agreement, respectively. We also examined the agreement between WB results and RT-PCR results using Cohen’s kappa for binary WB results and weighted kappa for graded WB results. In all cases the agreement was slight or poor. For anti-NP IgY WB results in relation to UGV-2 RT-PCR Cohen’s κ = -0.195 and κ(w) = -0.074; in relation to S5-like PT-PCR Cohen’s κ = 0.024 and κ(w) = 0.008; in relation to SMTV-2 RT-PCR Cohen’s κ = 0.088 and κ(w) = 0.03. For anti-NP IgM WB results in relation to UGV-2 RT-PCR Cohen’s κ = 0.067 and κ(w) = -0.024; in relation to S5-like RT-PCR Cohen’s κ = 0.061 and κ(w) = 0.02; in relation to SMTV-2 RT-PCR Cohen’s κ = 0.069 and κ(w) = 0.024.

**Table 7:** Results of the detection of IgY and IgM plasma antibodies against UGV-1 virions using WB in comparison to the diseases status (BIBD-positive or–negative, based on the presence of cytoplasmic IB in blood cells.

Western blotting		BIBD			Cohen’s κ	Sensitivity (95%CI)	Specificity (95%CI)
		+ve	-ve	Total			
WB UGV1 IgY	+ve	24	25	49	κ = 0.011 (-0.195–0.218)	70.59% (59.91–81.26)%	30.56% (19.76–41.35)%
	-ve	10	11	21			
WB UGV1 IgM	+ve	25	27	52	κ = -0.015 (-0.222–0.193)	73.53% (63.19–83.86)%	25.00% (14.86–35.14)%
	-ve	9	9	18			
Total		34	36	70			

#### Antibody detection by ELISA

Since the quantification of WB results is at best indicative of the antibody titres, we decided to set up an ELISA test for the detection of anti-reptarenavirus NP antibodies. We used purified UGV-1, recombinant UHV-1 NP, and the C-terminal portion of UHV-1 NP (UHV-1 NP-C) as the antigens.

#### ELISA results as quantitative variables

We examined the ELISA results against the BIBD status and the RT-PCR results using t-test. UGV-1 IgY ELISA OD values were significantly higher for BIBD- (p<0.001) and UGV-2 RT-PCR- (p<0.05) negative animals, whereas UGV-1 IgM ELISA OD values were significantly higher for BIBD-positive animals (p<0.05). UHV-1 NP IgY ELISA OD values were significantly higher for BIBD- (p<0.001) and UGV-2 RT-PCR- (p<0.01) negative animals, UHV-1 NP-C IgY ELISA OD values were significantly higher for BIBD (p<0.01) and UGV-2 RT-PCR (p<0.01) negative animals, and UHV-1 NP-C IgM ELISA OD values were significantly higher for BIBD- (p<0.05) and UGV-2 RT-PCR- (p<0.01) negative animals and for SMTV-2 RT-PCR-positive animals (p<0.05). Table 8 provides the detailed results of the analysis.

**Table 8:** ELISA results against RT-PCR and IB detection.

ELISA Alternative test	UGV1 IgY* (n) (95%CI)	UGV1 IgM* GM (n) (95%CI)	UHV1 NP IgY* (n) (95%CI)	UHV1 NP IgM* (n) (95%CI)	UHV-1 NP-C IgY* (n) (95%CI)	UHV-1 NP-C IgM* (n) (95%CI)
**BIBD +ve**	**0.155** (33) (0.095–0.252)	**0.561** (33) (0.479–0.657)	**0.156** (34) (0.114–0.213)	**0.250** (34) (0.202–0.308)	**0.251** (34) (0.191–0.329)	**0.290** (34) (0.241–0.351)
**BIBD -ve**	**0.553** (36) (0.337–0.906)	**0.448** (36) (0.399–0.503)	**0.306** (36) (0.244–0.385)	**0.255** (36) (0.199–0.327)	**0.556** (36) (0.452–0.682)	**0.379** (36) (0.339–0.422)
t-test	t = 3.7246, df = 67 **P<0.001**	t = -2.3586, df = 67 **p<0.05**	t = 3.5899, df = 68 **p<0.001**	t = 0.1294, df = 68 p = 0.903	t = 4.771, df = 68 **p<0.001**	t = 2.5368, df = 68 **p<0.05**
**UGV-2 RT-PCR +ve**	**0.209** (42) (0.133–0.329)	**0.464** (42) (0.398–0.540)	**0.173** (43) (0.132–0.226)	**0.254** (43) (0.214–0.301)	**0.282** (43) (0.221–0.361)	**0.296** (43) (0.254–0.344)
**UGV-2 PR-PCR -ve**	**0.530** (27) (0.288–0.975)	**0.464** (27) (0.458–0.596)	**0.326** (27) (0.251–0.123)	**0.249** (27) (0.179–0.346)	**0.598** (27) (0.480–0.744)	**0.402** (27) (0.356–0.454)
t-test	t = 2.5322, df = 67 **p<0.05**	t = -1.181, df = 67 p = 0.2118	t = 3.2325, df = 68 **p<0.01**	t = -0.1199, df = 68 p = 0.9049	t = 4.2732, df = 68 **p<0.001**	t = 2.9145, df = 68 **p<0.01**
**S5-like RT-PCR +ve**	**0.262** (60) (0.180–0.382)	**0.495** (60) (0.445–0.551)	**0.207** (61) (0.168–0.256)	**0.251** (61) (0.210–0.301)	**0.353** (61) (0.292–0.427)	**0.333** (61) (0.295–0.375)
**S5-like PT-PCR -ve**	**0.745** (9) (0.171–0.324)	**0.522** (9) (0.378–0.718)	**0.339** (9) (0.156–0.733)	**0.260** (9) (0.197–0.344)	**0.592** (9) (0.263–1.330)	**0.335** (9) (0.295–0.375)
t-test	t = 1.9239, df = 67 p = 0.0586	t = 0.3602, df = 67 p = 0.7199	t = 1.6353, df = 68 p = 0.1068	t = 0.1522, df = 68 p = 0.8795	t = 1.8398, df = 68 p = 0.0702	t = 0.041, df = 68 p = 0.9674
**SMTV-2 RT-PCR +ve**	**0.334** (53) (0.220–0.508)	**0.506** (53) (0.452–0.567)	**0.235** (54) (0.192–0.289)	**0.259** (54) (0.213–0.315)	**0.407** (54) (0.334–0.495)	**0.359** (54) (0.320–0.402)
**SMTV-2 PT-PCR -ve**	**0.212** (16) (0.088–0.507)	**0.475** (16) (0.383–0.589)	**0.178** (16) (0.098–0.546)	**0.230** (16) (0.179–0.296)	**0.292** (16) (0.171–0.500)	**0.258** (16) (0.198–0.337)
t-test	t = -1.0373, df = 67 p = 0.3033	t = -0.5349, df = 67 p = 0.5945	t = -1.1503, df = 68 p = 0.2541	t = -0.6136, df = 68 p = 0.5415	t = -1.4677, df = 68 p = 0.1468	t = -2.6477, df = 68 **p<0.05**
Total	0.301 (69) (0.207–0.436)	0.499 (69) (0.452–0.550)	0.221 (70) (0.180–0.270)	0.252 (70) (0.215–0.296)	0.377 (70) (0.312–0.456)	0.333 (70) (0.299–0.371)

*Optical density geometric mean

ELISA results for IgY and IgM from all the tests were analysed against population parameters and the other tests. At univariate level we used Analysis of Variance (ANOVA) to examine associations between age and antibody titres. UGV-1 IgY ELISA titres were the only ones significantly associated with age (F (6,59) = 3.52, p<0.01). Linear regression established that weight was significantly associated with ELISA titres for UGV-1 IgY and UGV-1 IgM (Regression equations UGV-1 IgY: F(1.67) = 32.4, R^2^ = 0.326, Predicted UGV-1 IgY = -1.245 + 1.556 weight; Predicted UGV-1 IgM: F(1.67) = 4.9 = -0.217–0.188 weight). There was no significant association between any of the ELISA test results and the animals’ sex. The results of the univariate analysis are presented in Table 9.

**Table 9:** Associations between ELISA results and population parameters, univariate analysis.

	Sex (95%CI)				Weight Linear regression results				Age ANOVA results	
OD geometric mean	Male	Female	All	p value	F R^2^	Coef	Adjusted *b* ((95%CI)	p value	F	p value
**UGV-1** **RT-PCR IgY**	**0.208** (0.122–0.352)	**0.422** (0.251–0.710)	**0.301** (0.207–0.436)	t = -1.9407, df = 67 p = 0.0565	(1.67) = 32.4 0.316	-0.245 (-1.532–0.959)	1.556 (1.010–2.102)	**p<0.0001**	(6,59) = 3.52	**p<0.01**
**UGV1** **RT-PCR IgM**	**0.540** (0.472–0.618)	**0.462** (0.400–0.534)	**0.499** (0.452–0.550)	t = 1.6002, df = 67 p = 0.1143	(1.67) = 4.90 0.0542	-0.217 (-0.304 - -0.131)	-0.188 (-0.357 –-0.185)	**P<0.05**	(6.59) = 1.26	p = 0.2876
**UHV-1 NP** **RT-PCR IgY**	**0.242** (0.196–0.299)	**0.202** (0.142–0.287)	**0.221** (0.180–0.270)	t = 0.8919, df = 68 p = 0.3756	(1.68) = 0.04 -0.141	-0.673 (-0.858–0.487)	0.036 (-0.319–0.391)	p = 0.84	(6,60) = 1	p = 0.4365
**UHV-1 NP** **RT-PCR IgM**	**0.265** (0.228–0.308)	**0.241** (0.181–0.320)	**0.252** (0.215–0.296)	t = 0.5993, df = 68 p = 0.5510	(1,68) = 0.43 -0.0083	-0.556 (-0.701 - -0.411)	-0.092 (-0.369–0.186)	p = 0.513	(6,60) = 0.71	p = 0.6398
**UHV-1 NPC** **RT-PCR IgY**	**0.382** (0.298–0.489)	**0.373** (0.276–0.504)	**0.377** (0.312–0.456)	t = 1.1234, df = 68 p = 0.9021	(1,68) = 0.86 -0.0021	-0.494 (-0.666 –-0.321)	0.153 (-0.178–0.485)	p = 0.358	(6,60) = 1.45	p = 0.2093
**UHV-1 NP-C** **RT-PCR IgM**	**0.335** (0.294–0.381)	**0.331** (0.277–0.396)	**0.333** (0 .299–0.371)	t = 0.1052, df = 68 p = 0.9165	(1,68) = 0.71 -0.0041	-0.514 (-0.312 –-0.416)	0.080 (-0.108–0.268)	p = 0.401	(6,60) = 0.77	p = 0.598

**Fig 3:**
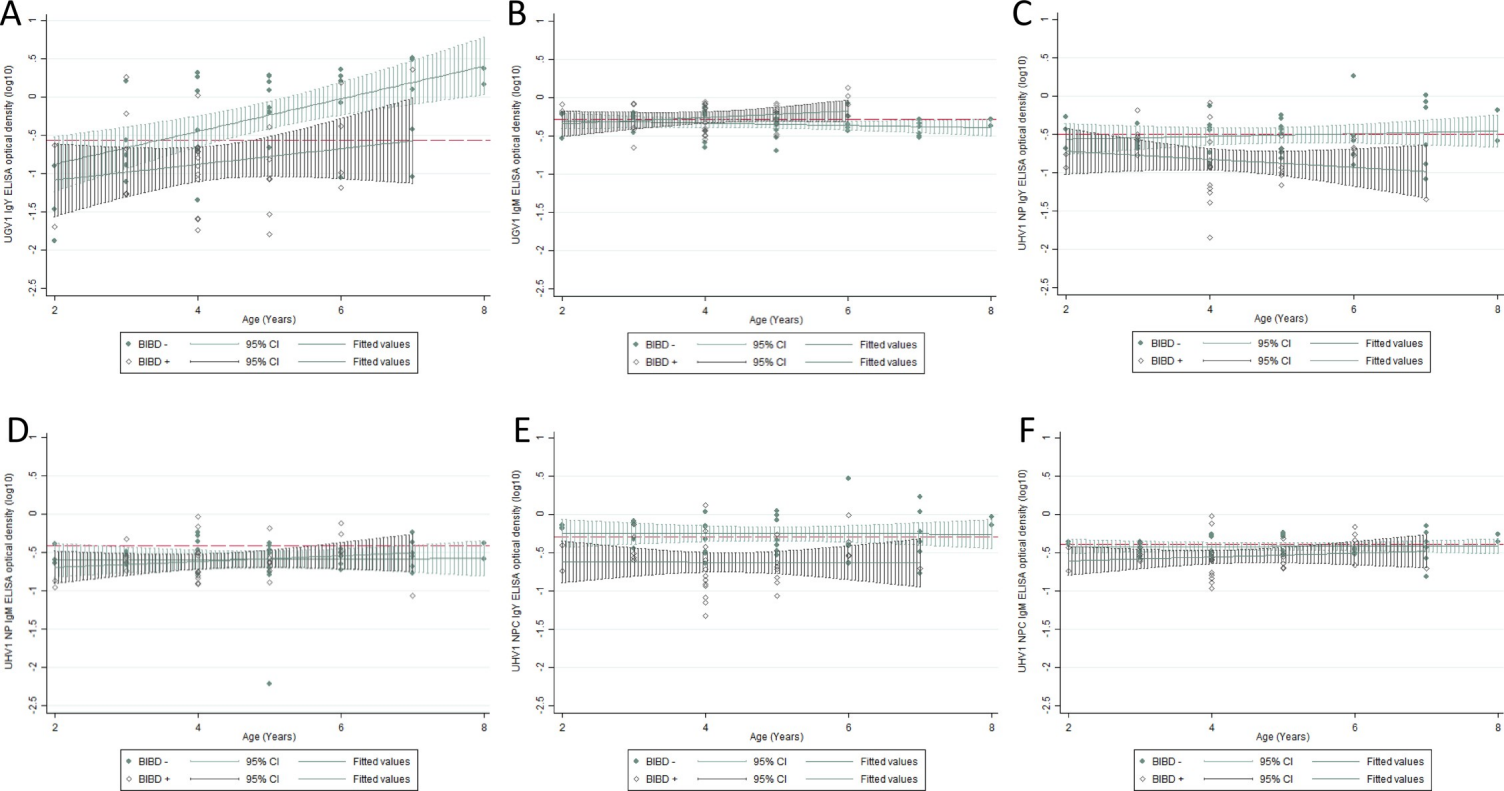
Associations of ELISA test results with age and BIBD status. A) UGV1 IgY, B) UGV1 IgM, C) UHV1 NP IgY, D) UHV1 NP IgM, E) UHV1 NPC IgY, F) UHV1 NPC IgM. The red lines indicate the ELISA cut-off point.

Using multivariable linear regression, we examined the associations of UGV-1 IgY and IgM with BIBD, weight and age. We established that both age and BIBD+ status were significantly associated with UGV-1 IgY antibody titres, F(2,63) = 16.94, and they accounted for 32.90% of antibody variability (p<0.001). The regression equation is: Predicted UGV- IgY OD(log10) = -1.147 + 0.181 age—0.4812 BIBD+. Fig 3A illustrates this association, with BIBD-negative animals demonstrating higher antibody titres than BIBD-positive ones. A similar model when fitted for UGV-1 IgM did not provide significant results. We include the graphic representation (Fig 3B) as the result may indicate an interesting trend of UGV-1 IgM remaining at higher levels for BIBD-positive animals because of continuous exposure from circulating virus while in BIBD-negative snakes, lack of such exposure may lead to UGV-1 IgM reduction in older animals. (Fig 3A–3F) demonstrates the association of all the ELISA test results with age and IB detection.

**Fig 4:**
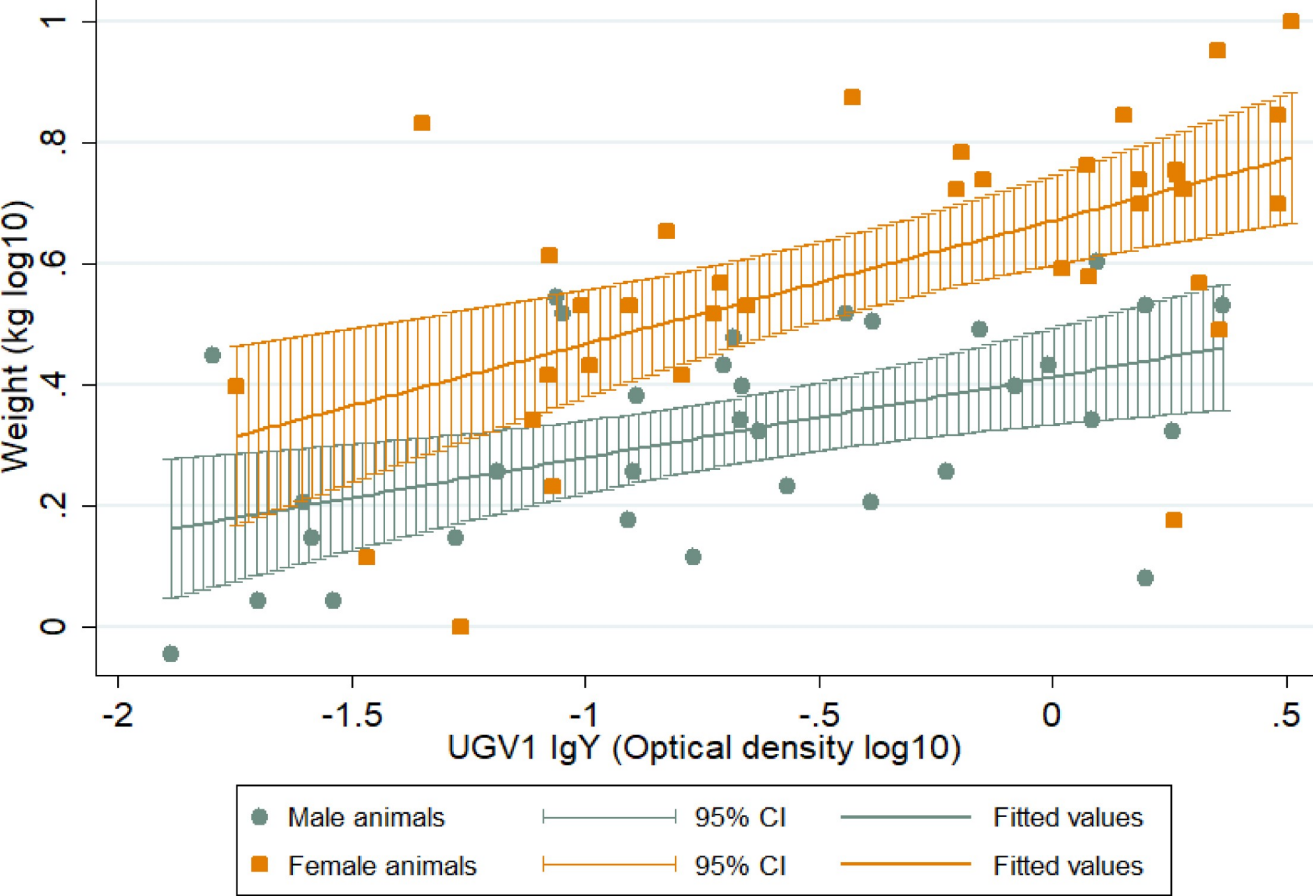
Association of body weight and UGV-1 IgY antibodies in female and male snakes.

Multivariable linear regression also established that age, sex and plasma UGV1 IgY were significantly associated (p<0.0001) with the weight of the animals, F(3,62) = 38.24 and they accounted for 63.22% of weight variability. The regression equation is: Predicted Weight = 0.079 + 0.075 age + 0.195 sex—0.096 UGV1 IgY OD. Fig 4 demonstrates this association separately for male and female animals. To establish linearity in this and all previous cases, we checked the residuals for normalcy using Shapiro-Wilk test and examined a residual versus fitted values plot.

We then investigated the potential association between the number of S segments found and the antibody response. Of the 23 BIBD-positive snakes in which all three viral S segments were detected, six (26.09%) were positive for anti-UGV IgY and 14 (63.64%) for anti-UGV IgM antibodies, four (18.18%) carried both IgY and IgM, and seven (31.82%) were negative for either antibodies. Among the nine snakes with two S segments were two (22.22%) that exhibited anti-UGV IgY antibodies, and three (33.33%) were positive for anti-UGV IgM antibodies. The two IgY-positive snakes also carried anti-NP IgM antibodies (22.22%); six snakes (66.67%) were negative for either antibodies. Both BIBD-positive snakes in which only the UGV-/S6-like S segment was detected exhibited an anti-NP IgM response; one also carried anti-NP IgY antibodies. All seven BIBD-negative animals tested positive for three viral S segments carried UGV-specific antibodies, five (71.43%) were IgY-positive, and three (42.86%) IgM-positive, one snake (14.29%) was positive for both Igs. Of the animals positive for two S segments (n = 23), the majority carried IgY (n = 14; 60.87%), nine (39.13%) were IgM-positive, and five (21.74%) were positive for both antibodies; five animals (21.74%) did not exhibit an antibody response. Both snakes in which a single viral S segment was detected exhibited both an IgY and an IgM response. Of the four RT-PCR negative animals, two (50%) showed a combined IgY and IgM response, one only had IgY antibodies, and one did not exhibit an anti-reptarenavirus response. There is no significant association between the number of segments and any of the ELISA results.

#### ELISA cut-off points

**Fig 5:**
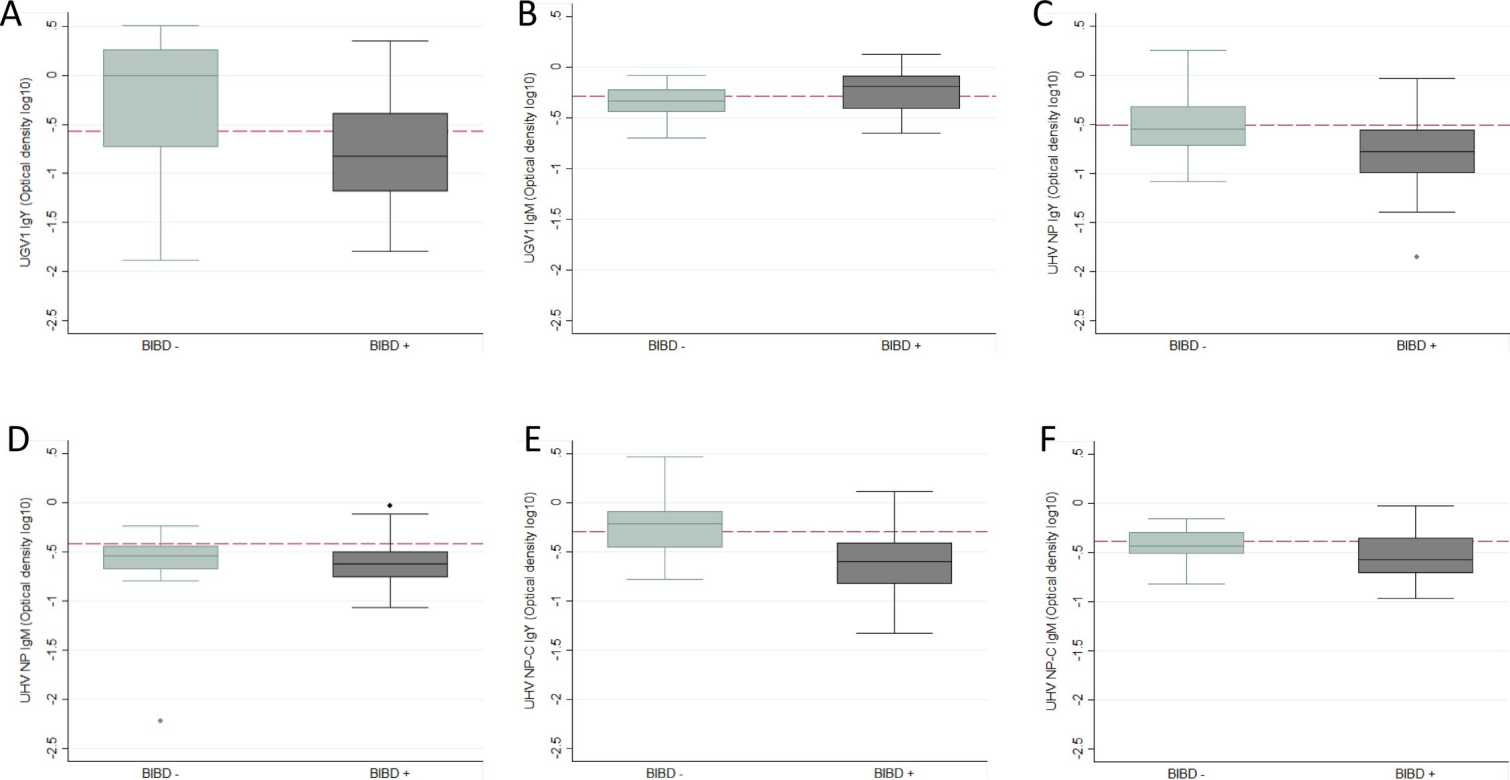
ELISA results including cut-off values for UGV-1 IgY and IgM, UHV NP IgY and IgM, UHV NP-C IgY and IgM antibodies in BIBD-positive and BIBD-negative snakes.

The background corrected raw ELISA data with cut-off values are presented in Fig 5. We tested the BIBD-positive snakes for the presence of anti-UGV-1 IgY and IgM antibodies and found nine (26.5%) IgY positives and 19 (57.58%) IgM positives of which seven (21.21%) were also IgY-positive. Thirteen animals (39.39%) did not exhibit any anti-UGV-1 antibodies (Table 10). Of the 36 BIBD-negative snakes 24 (66.67%) had anti-UGV-1 IgY and 16 (44.44%) anti-UGV-1 IgM antibodies, 10 animals (27.78%) showed both IgY and IgM; six snakes (16.67%) did not exhibit any anti-UGV-1 antibodies (Table 11).

**Table 10:** Results obtained from the examination of UGV-2, S5-like and TSMV-2 specific S-segments by RT-PCR and UGV-1, UHV-1 NP and UHV-1 NP-C specific IgY and IgM antibodies by ELISA—Animals with BIBD.

RT-PCR	UGV-1			UHV-1 NP			UHV-1 NP-C		
	IgY	IgM n = 33 tested	IgY and IgM n = 33 tested	IgY	IgM	IgY and IgM	IgY	IgM	IgY and IgM
Positive 34/34 (100%)	Pos 9/34 (26.47%)	Pos 19/33 (57.58%)	Pos 7/33 (21.21%)	Pos 6/34 (17.65%)	Pos 7/34 (20.59%)	Pos 4/34 (11.76%)	Pos 7/34 (20.59%)	Pos 10/34 (29.41%)	Pos 5/34 (14.71%)
	Neg 25/34 (73.53%)	Neg 14/33 (42.42%)	Neg 13/33 (39.39%)	Neg 28/34 (82.35%)	Neg 27/34 (79.41%)	Neg 25/34 (73.53%)	Neg 27/34 (79.41%)	Neg 24/34 (70.59%)	Neg 22/34 (64.71%)
3 Segments 23/34 (67.65%)	Pos 6/23 (26.09%)	Pos 14/22 (63.64%)	Pos 4/22 (18.18%)	Pos 2/23 (8.7%)	Pos 4/23 (17.39%)	Pos 1/23 (4.35%)	Pos 3/23 (13.04%)	Pos 7/23 (30.43%)	Pos 2/23 (8.7%)
	Neg 17/23 (73.91%)	Neg 8/22 (36.36%)	Neg 7/22 (31.82%)	Neg 21/23 (91.3%)	Neg 19/23 (82.61%)	Neg 18/23 (78.26%)	Neg 20/23 (86.96%)	Neg 16/23 (69.57%)	Neg 15/23 (65.22%)
2 Segments 9/34 (26.47%)	Pos 2/9 (22.22%)	Pos 3/9 (33.33%)	Pos 2/9 (22.22%)	Pos 3/9 (33.33%)	Pos 2/9 (22.22%)	Pos 2/9 (22.22%)	Pos 3/9 (33.33%)	Pos 2/9 (22.22%)	Pos 2/9 (22.22%)
	Neg 7/9 (77.78%)	Neg 6/9 (66.67%)	Neg 6/9 (66.67%)	Neg 6/9 (66.67%)	Neg 7/9 (77.78%)	Neg 6/9 (66.67%)	Neg 6/9 (66.67%)	Neg 7/9 (77.78%)	Neg 6/9 (66.67%)
1 Segment 2/34 (5.88%)	Pos 1/2 (50%)	Pos 2/2 (100%)	Pos 1/2 (50%)	Pos 1/2 (50%)	Pos 1/2 (50%)	Pos 1/2 (50%)	Pos 1/2 (50%)	Pos 1/2 (50%)	Pos 1/2 (50%)
	Neg 1/2 (50%)	Neg 0/2 (0%)	Neg 0/2 (0%)	Neg 1/2 (50%)	Neg 1/2 (50%)	Neg 1/2 (50%)	Neg 1/2 (50%)	Neg 1/2 (50%)	Neg 1/2 (50%)

Pos–positive; Neg—negative

**Table 11:** Results obtained from the examination of UGV-2, S5-like and TSMV-2 specific S-segments by RT-PCR and UGV-1, UHV-1 NP and UHV-1 NP-C specific IgY and IgM antibodies by ELISA—Animals without BIBD.

RT-PCR	UGV-1			UHV-1 NP			UHV-1 NP-C		
	IgY	IgM	IgY and IgM	IgY	IgM	IgY and IgM	IgY	IgM	IgY and IgM
Positive/ Negative 36/36 (100%)	Pos 24/36 (66.67%)	Pos 16/36 (44.44%)	Pos 10/36 (27.78%)	Pos 17/36 (47.22%)	Pos 9/36 (25%)	Pos 8/36 (22.22%)	Pos 19/36 (52.78%)	Pos 17/36 (47.22%)	Pos 16/36 (44.44%)
	Neg 12/36 (33.33%)	Neg 20/36 (55.56%)	Neg 6/36 (16.67%)	Neg 19/36 (52.78%)	Neg 27/36 (75%)	Neg 18/36 (50%)	Neg 17/36 (47.22%)	Neg 19/36 (52.78%)	Neg 16/36 (44.44%)
Positive 32/36 (88.89%)	Pos 21/32 (65.63%)	Pos 14/32 (43.75%)	Pos 8/32 (25.00%)	Pos 16/32 (50%)	Pos 9/32 (28.13%)	Pos 8/32 (25.00%)	Pos 17/32 (53.13%)	Pos 16/32 (50%)	Pos 15/32 (46.88%)
	Neg 11/32 (34.38%)	Neg 18/32 (56.25%)	Neg 5/32 (15.63%)	Neg 16/32 (50%)	Neg 23/32 (71.88%)	Neg 15/32 (46.88%)	Neg 15/32 (46.88%)	Neg 16/32 (50%)	Neg 14/32 (43.75%)
3 Segments 7/32 (21.88%)	Pos 5/7 (71.43%)	Pos 3/7 (42.86%)	Pos 1/7 (14.29%)	Pos 4/7 (57.14%)	Pos 2/7 (28.57%)	Pos 1/7 (14.29%)	Pos 3/7 (42.86%)	Pos 3/7 (42.86%)	Pos 3/7 (42.86%)
	Neg 2/7 (28.57%)	Neg 4/7 (57.14%)	Neg 0/7 (0%)	Neg 3/7 (42.86%)	Neg 5/7 (71.43%)	Neg 2/7 (28.57%)	Neg 4/7 (57.14%)	Neg 4/7 (57.14%)	Neg 4/7 (57.14%)
2 Segments 23/32 (71.88%)	Pos 14/23 (60.87%)	Pos 9/23 (39.13%)	Pos 5/23 (21.74%)	Pos 11/23 (47.83%)	Pos 6/23 (26.09%)	Pos 6/23 (26.09%)	Pos 13/23 (56.52%)	Pos 12/23 (52.17%)	Pos 11/23 (47.83%)
	Neg 9/23 (39.13%)	Neg 14/23 (60.87%)	Neg 5/23 (21.74%)	Neg 12/23 (52.17%)	Neg 17/23 (73.91%)	Neg 12/23 (52.17%)	Neg 10/23 (43.48%)	Neg 11/23 (47.83%)	Neg 9/23 (39.13%)
1 Segment 2/32 (6.25%)	Pos 2/2 (100%)	Pos 2/2 (100%)	Pos 2/2 (100%)	Pos 1/2 (50%)	Pos 1/2 (50%)	Pos 1/2 (50%)	Pos 1/2 (50%)	Pos 1/2 (50%)	Pos 1/2 (50%)
	Neg 0/2 (0%)	Neg 0/2 (0%)	Neg 0/2 (0%)	Neg 1/2 (50%)	Neg 1/2 (50%)	Neg 1/2 (50%)	Neg 1/2 (50%)	Neg 1/2 (50%)	Neg 1/2 (50%)
Negative 4/36 (11.11%)	Pos 3/4 (75%)	Pos 2/4 (50%)	Pos 2/4 (50%)	Pos 1/4 (25%)	Pos 0/4 (0%)	Pos 0/4 (0%)	Pos 2/4 (50%)	Pos 1/4 (25%)	Pos 1/4 (25%)
	Neg 1/4 (25%)	Neg 2/4 (50%)	Neg 1/4 (25%)	Neg 3/4 (75%)	Neg 4/4 (100%)	Neg 3/4 (75%)	Neg 2/4 (50%)	Neg 3/4 (75%)	Neg 2/4 (50%)

Pos–positive; Neg—negative

Within the group of BIBD-positive snakes were six (17.65%) that carried anti-UHV-1-NP IgY and seven (20.59%) positive for IgM. Four snakes (11.76%) carried both antibodies and 25 (73.53%) did not exhibit any anti-UHV-1 antibodies. The examination of UHV-1-NP antibodies in the BIBD-negative group identified 17 snakes (47.22%) with IgY and nine (25%) with IgM antibodies. A combination of IgY and IgM was detected in eight snakes (22.22%), whereas 18 (50%) were negative for both anti-UHV-1-NP antibodies. Of the BIBD-positives snakes seven (20.59%) had anti-UHV-1-NP-C IgY and 10 (29.41%) IgM antibodies. Both antibodies were found in five snakes (14.71%) and 22 (64.71%) were negative for IgY and IgM. Among the BIBD-negative animals 19 (52.78%) carried IgY and 17 (47.22%) were positive for IgM of which 16 (44.44%) also exhibited an IgY antibody response; 16 snakes (44.44%) did not carry any anti-UHV-1-NP-C antibodies.

We examined the agreement of the different ELISA tests with the BIBD status using Cohen’s kappa (Table 11). Because significantly more BIBD-positive animals were testing negative for IgY (above the cut-off point, see Table 1), and because the measured OD values in ELISA were lower in BIBD-positive than in BIBD-negative animals we calculated the test agreement, using Cohen’s kappa, considering negative ELISA results equivalent to positive BIBD ones. We reversed thus the UGV-1 IgY ELISA results (positive to negative) which led to a moderate agreement with BIBD (κ = 0.429). The same applied to UHV-1 NP IgY ELISA (κ = 0.293) and UHV NP-C IgY (κ = 0.319) which showed fair agreement with BIBD. All IgM ELISA results show slight or poor agreement with BIBD (UGV-1 IgM, κ = 0.131; UHV-1 NP IgM, κ = -0.045; UHV1 NP-C IgM, κ = -0.179). Results are summarised in Tables 12 and 13 including the agreement between ELISA results and RT-PCR. All results indicate poor to fair agreement between tests. 95% confidence intervals were calculated for Cohen’s kappa and further confirm the lack of agreement between tests [42].

**Table 12:** ELISA results based on the cut-off points against inclusion detection including test agreement and sensitivity/Specificity.

ELISA test		BIBD			Cohen’s κ (95%CI)	Sensitivity	Specificity
		+ve	-ve	Total			
UGV1 IgY	+ve	25	11	34	κ = 0.429 (0.213–0.645)	73.53% (63.19–83.86) %	69.44% (58.65–80.24) %
	-ve	9	25	36			
UGV1 IgM*	+ve	14	20	34	κ = -0.131 (-0.360–0.097)	42.42% (30.76–54.09) %	44.44% (32.72–56.17) %
	-ve	19	16	35			
UHV1 NP IgY	+ve	28	19	47	κ = 0.293 (0.075–0.510)	82.35% (73.42–91.28) %	47.22% (35.53–58.92) %
	-ve	6	17	23			
UHV1 NP IgM	+ve	7	9	16	κ = 0.043 (-0.145–0.232)	79.41% (69.94–88.88) %	25.00% (14.86–35.14) %
	-ve	24	27	54			
UHV1 NP-C IgY	+ve	27	17	44	κ = 0.319 (0.100–0.539)	79.41% (69.94–88.88) %	52.78% (41.08–64.47) %
	-ve	7	19	26			
UHV1 NP-C IgM	+ve	24	19	43	κ = 0.177 (-0.051–0.405)	70.59% (59.91–81.26) %	47.22% (35.53–58.92) %
	-ve	10	17	27			
Total		34	36	70			

*missing value

**Table 13:** Agreements of ELISA tests with IB detection and RT-PCR.

ELISA test		BIBD			UGV-2			S5-like			SMTV-2			
		+ve	-ve	Cohen’s κ	+ve	-ve	Cohen’s κ (95%CI)	+ve	-ve	Cohen’s κ	+ve	-ve	Cohen’s κ	Total
UGV1 IgY	+ve	25	11	κ = 0.429 (0.213–0.645)	28	8	0.339 (0.119–0.558)	34	2	0.153 (-0.009–0.316)	26	10	-0.103 (-0.307–0.102)	36
	-ve	9	25		15	19		27	7		28	6		34
UGV1 IgM*	+ve	14	20	κ = -0.131 (-0.360–0.097)	21	13	0.018 (-0.209–0.244)	30	4	0.025 (-0.134–0.184)	25	9	-0.064 (-0.267–0.139)	34
	-ve	19	16		21	14		30	5		28	7		35
UHV1 NP IgY	+ve	28	19	κ = 0.293 (0.075–0.510)	33	14	0.256 (0.018–0.494)	42	5	0.080 (-0.129–00289)	35	12	-0.088 (-0.307–0.131)	47
	-ve	6	17		10	13		19	4		19	4		23
UHV1 NP IgM	+ve	27	27	κ = 0.043 (-0.145–0.232)	35	19	0.119 (-0.103–0.342)	47	7	-0.005 (-0.220–0.209)	40	14	-0.134 (-0.333–0.064)	54
	-ve	7	9		8	8		14	2		14	2		16
UHV1 NP-C IgY	+ve	27	17	κ = 0.319 (0.100–0.539)	33	11	0.363 (0.133–0.592)	40	4	0.117 (-0.083–0.318)	33	11	-0.063 (-0.284–0.159)	44
	-ve	7	19		10	16		21	5		21	5		26
UHV1 NP-C IgM	+ve	24	19	κ = 0.177 (-0.051–0.405)	31	12	0.276 (0.037–0.515)	37	6	-0.032 (-0.209–0.144)	30	13	-0.207 (-0.400 –-0.014)	43
	-ve	10	17		12	15		24	3		24	3		27
Total		34	36		43	27		61	9		54	16		70

*missing value

Using univariate analysis, we examined the ELISA test results based on the cut-off points for associations with population parameters. There is no significant association between animal sex and any of the ELISA results. The presence of UGV-IgY is significantly associated with weight. The geometric mean (GM) weight of UGV-IgY-positive animals (n = 34) is 3.809 kg (95% CI: 3.159–4.594) while for UGV-IgY-negative animals (n = 36) the geometric mean weight is 2.193 kg (95%CI: 1.858–2.589kg, p<0.0001). This association remained significant after stratification for sex for both male and female animals (Male: UGV-IgY positive animals (n = 14) GM = 2.448 kg [95%CI: 1.995–3.004], UGV-IgY negative animals (n = 20) GM = 1.809 kg [95%CI: 1.484–2.206], p<0.05; Female: UGV-IgY positive animals (n = 16) GM = 5.192 kg [95%CI: 4.283–6.293 ], UGV-IgY negative animals (n = 20) GM = 2.788 kg [95%CI: 2.167–3.588], p<0.001). A significant association was also identified between UGV-IgY and the animals’ age. UGV-IgY-positive animals are significantly older than negative animals (p<0.001). The average age is 5.313 years (95%CI: 4.783–5.842) and 4 years (95%CI: 3.567–4.329) for UGV-IgY positive animals (n = 32) and negative animals (n = 35) respectively. After stratifying for sex, the association remained significant for female animals (UGV1 IgY positive animals (n = 20) mean age = 5.5 years) [95%CI: 4.865–6.135]; UGV-IgY negative (n = 16) mean age = 4.063 years [95%CI: 3.568–4.557], p<0.005). No other association was identified between any of the ELISA results based on the cut-off point and population parameters. All the results are presented in Tables 14 and 15.

**Table 14:** IgY ELISA cut-off point results against population parameters, univariate analysis including stratification by sex.

	Sex (Row%) (Col%)			Weight* (95% CI) N = 70			Age**(n) (95% CI) N = 67		
	**M**	**F**	**Total**	**M**	**F**	**Total**	**M**	**F**	**Total**
**UGV- 1** **IgY ELISA** **-ve**	**20** (55.56%) (58.82%)	**16** (44.44%) (44.44%)	**36** (100.00%) (51.43%)	**1.809** (1.484–2.206)	**2.788** (2.167–3.588)	**2.193** (1.858–2.589)	**3.947** (19) (3.220–4.674)	**4.063** (16) (3.568–4.557)	**4.000** (35) (3.567–4.329)
**UGV-1** **IgY ELISA** **+ve**	**14** (41.18%) (41.18%)	**20** (58.82%) (55.86%)	**34** (100.00%) (48.57%)	**2.448** (1.995–3.004)	**5.192** (4.283–6.293)	**3.809** (3.159–4.594)	**5.000** (12) (3.951–6.049)	**5.500** (20) (4.865–6.135)	**5.313** (32) (4.783–5.842)
	χ^2^ = 1.4473, p = 0.229			t = -2.1855, df = 32 **p<0.05**	t = -4.2166, df = 34 **p<0.001**	t = -4.480, df = 68 **p<0.0001**	t = -1.8251, df = 29 p = 0.0783	t = -3.609, df = 34 **p<0.005**	t = -3.935, df = 65 **p<0.001**
**UHV-1 NP** **IgY ELISA** **-ve**	**23** (48.94%) (67.65%)	**24** (51.06%) (66.67%)	**47** (100.00%) (67.14%)	**2.005** (1.666–2.412)	**3.711** (2.930–4.701)	**2.745** (2.313–3.259)	**4.500** (22) (3.819–5.181)	**4.750** (24) (4.163–5.337)	**4.630** (46) (3.865–5.373)
**UHV-1 NP** **IgY ELISA** **+ve**	**11** (47.83%) (32.35%)	**12** (52.17%) (33.33%)	**23** (100.00%) (32.86%)	**2.145** (1.622–2.837)	**4.436** (3.268–6.020)	**3.134** (2.442–4.020)	**4.000** (9) (2.562–5.438)	**5.083** (12) (4.207–5.959)	**4.619** (21) (3.865–5.374)
	χ^2^ = 0.076, p = 0.930			t = -0.4349, df = 32 p = 0.6667	t = -0.9422, df = 34 p = 0.3527	t = -0.894, df = 68 p = 0.3746	t = 0.773, df = 29 p = 0.4458	t = -0.6797, df = 34 p = 0.5013	t = 0.285, df = 65 p = 9774
**UHV-1 NP-C** **IgY ELISA** **-ve**	**21** (47.73%) (61.76%)	**23** (52.27%) (63.89%)	**44** (100.00%) (62.86%)	**2.121** (1.745–2.576)	**3.861** (3.086–4.831)	**2.901** (2.446–3.440)	**4.526** (19) (3.784–5.269)	**4.739** (23) (4.265–5.213)	**4.643** (42) (4.237–5.049)
**UHV-1 NP-C** **IgY ELISA** **+ve**	**13** (50.00%) (38.24%)	**13** (50.00%) (36.11%)	**26** (100.00%) (37.14%)	**1.938** (1.508–2.492)	**4.079** (2.870–5.798)	**2.812** (2.186–3.618)	**4.083** (12) (2.951–5.215)	**5.077** (13) (3.989–6.165)	**4.600** (25) (3.846–5.354)
	χ2 = 0.0338, p = 0.854			t = 0.6009, df = 32 p = 0.5521	t = -0.2922, df = 34 p = 0.7718	t = 0.2146, df = 68 p = 0.831	t = 0.7342, df = 29 p = 0.4687	t = -0.7021, df = 34 p = 0.4874	t = 0.112, df = 35 p = 0.914
**Total**	**34** (48.57%) (100.00%)	**36** (51.43%) (100.00%)	**70** (100.00%) (100.00%)	**2.049** (1.770–2.372)	**3.938** (3.287–4.719)	**2.867** (2.497–3.293)	**4.355** (31) (3.759–4.950)	**4.861** (36) (4.395–5.327)	**4.626** (67) (4.260–4.994)

*Kg, geometric mean

**Mean Years

**Table 15:** IgM ELISA cut-off point results against population parameters, univariate analysis including stratification by sex.

	**Sex** (Row%) (Col%)			Weight* (95% CI) UGV-1: N = 69; UHV- NP, UHV-1 NP-C: N = 70			Age**(n) (95% CI) UGV-1: N = 66; UHV-1 NP, UHV-1 NP-C: N = 67		
	**M**	**F**	**Total**	**M**	**F**	**Total**	**M**	**F**	**Total**
**UGV- 1** **IgM ELISA** **-ve**	**14** (41.18%) (41.18%)	**20** (58.82%) (57.14%)	**34** (100.00%) (49.28%)	**2.623** (2.204–3.120)	**3.771** (2.867–4.962)	**3.248** (2.712–3.889)	**5.000** (13) (3.983–6.017)	**4.750** (20) (4.145–5.355)	**4.848** (33) (4.338–5.359)
**UGV-1** **IgM ELISA** **+ve**	**20** (57.14%) (58.82%)	**15** (42.86%) (42.86%)	**35** (100.00%) (50.72%)	**1.724** (1.424–2.087)	**3.949** (3.089–5.048)	**2.459** (2.008–3.011)	**3.889** (18) (3.167–4.610)	**4.867** (15) (4.060–5.674)	**4.333** (33) 3.797–4.870)
**Total**	**34** (49.28%) 100.00%)	**35** (50.72%) (100.00%)	**69** (100.00%) (100.00%)	**2.049** (1.770–2.372)	**3.938** (3.287–4.719)	**2.820** (2.461–3.233)	**4.355** (31) (3.759–4.950)	**4.800** (35) (4.338–5.262)	**4.595** (66) (4.225–5.957)
	χ^2^ = 1.7590, p = 0.185			t = 3.2678, df = 32 **p<0.01**	t = -0.2540, df = 33 p = 0.818	t = 2.0827, df = 67 **p<0.05**	t = 1.968, df = 29 p = 0.0578	t = -0.2502, df = 33 p = 0.8040	t = 1.417, df = 67 p = 0.161
**UHV-1 NP** **IgM ELISA** **-ve**	**27** (50.00%) (79.41%)	**27** (50.00%) (75.00%)	**54** (100.00%) (77.14%)	**2.042** (1.757–2.374)	**3.841** (3.109–4.746)	**2.801** (2.404–3.263)	**4.280** (25) (3.633–4.927)	**4.778** (27) (4.237–5.319)	**4.538** (52) (4.128–4.949)
**UHV-1 NP** **IgM ELISA** **+ve**	**7** (43.75%) (20.59%)	**9** (56.25%) (25.00%)	**16** (100.00%) (22.86%)	**2.075** (1.212–3.553)	**4.245** (2.764–6.521)	**3.104** (2.183–4.413)	**4.667** (6) (2.603–6.730)	**5.111** (9) (3.994–6.228)	**4.629** (15) (4.034 (4.260–4.994)
	χ^2^ = 0.1930, p = 0.660			t = -0.0881, df = 32 p = 0.9304	t = -0.4807, df = 34 p = 0.6338	t = -0.6185, df = 68 p = 0.538	t = -0.5174, df = 29 p = 0.6088	t = -0.6237, df = 34 p = 0.5370	t = -0.893, df = 65 p = 0.375
**UHV-1 NP-C** **IgM ELISA** **-ve**	**21** (48.84%) (61.76%)	**22** (51.16%) (61.11%)	**43** (100.00%) (61.43%)	**2.155** (1.812–2.564)	**3.754** (3.003–4.602)	**2.863** (2.436–3.364)	**4.350** (20) (3.617–5.083)	**4.682** (22) (4.200–5.163)	**4.524** (42) (4.109–4.938)
**UHV-1 NP-C** **IgM ELISA** **+ve**	**13** (48.15%) (38.24%)	**14** (51.85%) (38.89%)	**27** (100.00%) (38.57%)	**1.888** (1.414–2.521)	**4.247** (3.018–5.978)	**2.875** (2.204–3.750)	**4.364** (11) (3.152–5.576)	**5.143** (14) (4.134–6.152)	**4.800** (25) (4.065–4.994)
	χ^2^ = 0.0032, p = 0.955			t = 0.8911, df = 32 p = 0.3795	t = -0.6709, df = 34 p = 0.5068	t = -0.295, df = 68 p = 0.797	t = -0.022, df = 29 p = 0.9826	t = -0.9792, df = 34 p = 0.3344	t = -0.723, df = 65 p = 0.472
**Total**	**34** (48.57%) (100.00%)	**36** (51.43%) (100.00%)	**70** (100.00%) (100.00%)	**2.049** (1.770–2.372)	**3.938** (3.287–4.719)	**2.867** (2.497–3.293)	**4.355** (31) (3.759–4.950)	**4.861** (36) (4.395–5.327)	**4.626** (67) (4.260–4.994)

*Kg, geometric mean

**Mean Years

## Discussion

In this study, we investigated the association between BIBD, pathogen detection, population parameters and serological findings in a cohort of snakes from one breeding colony. As our previous studies had implied an association between BIBD and low antibody levels [7,14], the main focus of this study was on a potential link between anti-reptarenavirus antibody levels and BIBD. We hypothesised that some reptarenavirus S segments can be found more frequently in snakes with BIBD, and that healthy and diseased snakes would show different S segment profiles. We examined a panel of 70 blood samples, evenly distributed by sex, collected on the same day from the entire animal cohort. Because snakes are poikilotherms, we considered minimising the environmental influence on the immune response to be essential. Therefore, the study was restricted to a single breeding colony where animals are kept under virtually the same husbandry conditions with regards to moisture, light, feeding regime and temperature, except that male snakes are kept at 2–5°C lower temperatures than females to increase reproductive activity.

We started by dividing the sample panel in BIBD positives and negatives based on the detection of IBs in blood cells, using blood smears stained under quality controlled conditions. We used the presence of IBs in combination with confirmed reptarenavirus infection as the diagnostic criteria for BIBD, since we consider it likely that the presence of reptarenavirus NP in the form of IBs in cells will eventually result in clinical signs and death of affected animals [1,12,13]. The examination of population parameters in our study did not show an association of age and the presence of IB, suggesting that the time and duration of the infection would not be a factor in the development of BIBD, though this is highly speculative as data on, for example, the introduction of individual animals was not available. Also, a dependency of sex and BIBD could not be shown, but we could demonstrate a statistically significant association between BIBD and reduced body weight in female snakes. While this may reflect the low number of snakes included in the study, it might also be indicative of metabolic or behavioural changes in the infected snakes. Since reptarenavirus replication is temperature sensitive [43], one could also speculate that the viruses replicate more efficiently in female snakes as these are housed at slightly higher temperatures. Further studies on the optimal reptarenavirus replication temperature would be required to address this hypothesis.

By NGS and de novo genome assembly, we identified two pairs of hartmanivirus L and S segments, several reptarenavirus L segments but only a single reptarenavirus S segment (UGV-like) from the RNA of a BIBD-positive blood pool [10]. Interestingly, reads matching reptarenaviruses were clearly less abundant in the RNA sample extracted from the BIBD-negative blood. This finding could indicate higher replication or more intense viraemia in the BIBD-positive snakes, however, it could also be explained by unknown factors related to NGS library preparation. As we aimed to study the immune response using NP as the antigen, we used the S segment primers of our previous study [1] in RT-PCRs to screen the pools, and identified two additional S segments (S5-like and TSMV-2) within the pools. Screening of all individual samples for UGV-like, S5-like, and TSMV-2 S segments by RT-PCR showed that 97.1% of the BIBD-positive snakes carried the UGV-like S-segment. This observation is well in line with previous studies, in which we [1,11] and others [12] have observed that UGV-/S6-like S segments are often found in snakes with BIBD. In contrast, we found the UGV-/S6-like S segment only in 27.8% of the BIBD-negative snakes. As the mechanisms behind IB formation are still unknown, one could speculate that UGV-/S6-like NP would be more prone to IB formation. However, in our first report on reptarenaviruses in snakes, we purified IBs from infected cell cultures and used peptide mass fingerprinting to identify the main protein component as University of Helsinki virus-1 (UHV-1) NP. This finding suggests that IB formation is similar between different reptarenavirus species (or S segments). Thus one explanation on why UGV-/S6-like S segments are often found in snakes with BIBD could instead lie in the GPC that is also carried in the S segment. The origin and reservoir host(s) of reptarenaviruses remain unknown, however, it seems obvious that UGV-/S6-like GPC allows the virus to spread efficiently among boas. As IBs are found in various tissues, the UGV-/S6-like GPC could also allow wide tissue tropism. Our findings indicated that detection of UGV-/S6-like S segment had the closest substantial agreement (κ = 0.6878) with BIBD. However, further work will be required to establish the sensitivity and specificity of UGV-/S6-like S segment detection in BIBD diagnosis.

The reptile immune response is not known in great detail, and its description is often subjected to a comparison with the mammalian immune system. It is also unclear how much immune response mechanisms vary within the class Reptilia or even within the clade Ophidia inside the order Squamata since studies on the immune response of snakes partially report controversial findings, for instance regarding the increase in titres after repeated antigen exposure in colubrid snakes [40]. Also, different IgY isotypes of certain snake species have been described [37], and a secretory immunoglobulin has only been found in the bile of the northwestern garter snake (*Thamnophis ordinoides*) [44]. The fact that we studied samples collected at a single time point from naturally infected snakes for which the time of infection was unknown, made the evaluation of antibody response kinetics impossible. However, the analysis of IgY and IgM antibodies by WB and ELISA showed that the presence of anti-UGV NP IgY is negatively correlated to the presence of IB and thereby BIBD (Fig 2). Although GPC and NP are encoded by the S segment, it remains to be studied whether GPC induces a similar immune response. We could not detect anti-GPC antibodies by WB, however, the result most likely reflects lack of sensitivity rather than lack of antibodies since we used purified virions (the NP is by far the most prominent protein in the virion) as the antigen. Further evidence of a possible association between infection with a virus bearing UGV-/S6-like S segment and BIBD is the observation that we found a significant positive association between weight and plasma UGV1 IgY titres. The observed variable occurrence of IgY and IgM antibodies in individual snakes could be due to the prolonged persistence of IgM and the variable onset of IgY production [21,39]. Anti-UGV NP IgM antibody titres showed a trend to lower in the older BIBD-negative snakes, which could reflect exhaustion of the immune system or a gradual class switch towards IgY. The current knowledge on the role of IgM and its age dependency in protective immunity in snakes is scarce. Natural antibodies (NAbs) are thought to compensate the decreasing sensitivity of the adaptive immune system in ageing snakes [45]. Interestingly, NAbs are also suggested to provide protection against mammarenavirus (LCMV) infection by epitope recognition [46].

The timing of infection greatly influences the immune response, as shown for LCMV, the prototypic arenavirus. Exposure *in utero* or as a neonate results in chronic infections [1,47]. Persistently infected LCMV carriers were thought to develop a state of tolerance, accepting the virus as endogenous, and therefore do not respond by antibody production [48]. However, later studies demonstrated an immunological response towards LCMV and concluded that low antibody levels were due to the formation of immune complexes that were deposited in the glomeruli of the kidney [49]. Further studies are needed to demonstrate whether such immune complexes are present in snakes with BIBD and/or in snakes infected with reptarenaviruses *in utero* or as neonates. Several studies also elucidated a dependency of antibody production on different strains of viruses and mice and a different IgG isotype profile in chronic vs. acute murine infections [50]. These antibody profiles were attributed to involvement of different T cell populations in acute and chronic infections, and associated with varying clinical signs [50]. Extensive studies by Oldstone and colleagues with the LCMV Armstrong 53b strain (ARM) as the parental virus demonstrated the emergence of virus variants with varying tissue tropism in mice [51]. Infection with the parental ARM isolate induced a strong CD8^+^ T cell response, while the CD8^+^ T cell response was aborted in mice infected with clone 13 (Cl 13) isolated from lymphoid cells of neonate mice infected with ARM [51]. LCMV strains and variants with high affinity for α-dystroglycan (e.g. Cl 13), the cellular receptor for Old World mammarenaviruses [52], can enter dendritic cells (DCs) [51]. Infected DCs can then be destroyed by the antiviral CD8^+^ T cell response [53] or remain functionally impaired [51]. The loss of the DC function as professional antigen presenting cells significantly contributes to the overall immunosuppression seen as a consequence of LCMV infection [51]. The receptor and the ability of reptarenaviruses to infect DCs are currently unknown. However, like LCMV [54], reptarenaviruses infect lymphoid cells [55], and could thus use immunosuppression mechanisms similar to those employed by LCMV. One could also speculate that the swarm of S segments often found in snakes with BIBD would contribute to immunosuppression by enabling a broader cell tropism for the virus.

Another aspect of LCMV induced immunosuppression is the exhaustion of CD4^+^ and CD8^+^ T cells that occurs in chronically LCMV infected mice [56,57]. Furthermore, the functional impairment of CD4^+^ T cells negatively influences the antibody response [56,57]. Also, the exhaustion of CD4^+^ T cells reduces the production of antibodies, as demonstrated by providing virus-specific CD4^+^ T cells from transgenic mice to chronically infected animals [56]. Mice persistently infected with LCMV do not possess LCMV-specific CD8^+^ T cells [50], and CD4^+^ T cells are absent in transplacentally infected mice [57]. The attenuation of T cell dependent immune functions as well as immune complex formation support the assumption that animals infected via vertical transmission show lower antibody levels than horizontally infected animals. It is possible that vertical transmission also occurs for maternal antibodies in ovoviviparous snakes, such as *B*. *constrictor*. This could theoretically compensate for the embryo’s immunological incompetence; however, how this aligns with the fact that persistently infected mothers pass both their reptarena- [1] and hartmaniviruses [10] to the newborn is not clear. Many snakes examined in the present study are related, as they represent a breeding colony; therefore, it is not possible to determine how many were horizontally infected. It is tempting to speculate that the snakes with high antibody titres were horizontally infected, whereas the BIBD-positive animals with low antibody titres were vertically infected. This would tie in with observations on LCMV which leads to reduced levels of IgG2a subclass in persistently infected mice [50]. LCMV Cl 13 can induce persistent infection, which results in exhaustion of virus-specific T cells and is associated with generalized immunosuppression in adult mice [51]. Something similar could occur during reptarenavirus infection. It is possible that there are reptarenavirus S segments with point mutations, similar to that in LCMV Cl 13 that alter the cell tropism and contribute to immunosuppression. Alternatively, multiple S segments could allow infection of different subsets of lymphoid cells, thus resulting in immunosuppression similar to that of LCMV Cl 13. In addition to the antibody and T cell responses, reptarenaviruses can be expected to influence the innate immune system in a manner similar to that of mammarenaviruses, i.e. via inhibition of type I interferon production [10,17,18,58]. Indeed, a general reptarenavirus-induced immunosuppression would tie in with the increased incidence of bacterial infections and/or neoplastic processes in snakes with BIBD [2–4].

This is to our knowledge the first report to thoroughly assess the adaptive immune response of boid snakes towards reptarenaviruses. By characterising a single breeding collection, we could demonstrate that one individual virus, UGV-/S6-like S segment, was strongly associated with BIBD. Supporting the link between the presence of UGV-/S6-like S segment and BIBD, we found a negative correlation between BIBD and the presence of anti-UGV NP antibodies. Future studies, either longitudinal or experimental infection driven, are needed to understand the kinetics of the antibody response in snakes with reptarenavirus infection. Our results do, however, suggest that presence/absence of UGV-/S6-like S segment RNA and presence/absence of anti-UGV NP IgY antibodies could serve to a limited extent in the *ante mortem* diagnostics of BIBD.

## Materials and methods

### Study cohort and samples, cytological examination

We studied a breeding collection of 70 *Boa constrictor* snakes comprising 36 female and 34 male adult individuals, aged between two and eight years (Table 1). Husbandry conditions included humidity of approximately 60% and a season-dependent light regime with photoperiods of 12–13 hours during warm and 9–10 hours during cold months. Female snakes were kept at an environmental temperature of 26–33°C with a drop of 3–4°C during night, but not deceeding 24°C whereas the males were kept at an environmental temperature approximately 2–5°C lower than the females with a minimum temperature of 23°C The cohort included two debilitated snakes (one male, animal 1.20; one female, animal 1.29) and one female snake with cloacal prolapse (animal 1.18); the remaining animals were clinically healthy. In June 2015, one snake from the collection had been euthanised due to clinical signs, and post mortem examination had confirmed BIBD diagnosis. Subsequent analysis of blood samples from 14 snakes had revealed the presence of cytoplasmic IBs in blood cells of eight snakes, confirmed that they also suffered from BIBD. These findings prompted the owner to have the entire breeding colony tested for BIBD a year later. In July 2016, blood samples were collected in 1.3 ml K3E EDTA tubes (Sarstedt) by either caudal tail vein venipuncture or cardiocentesis. All snakes were weighed before bleeding. No ethical permissions were required for these diagnosis-motivated blood samplings.

### Blood samples and smears

Cytological examination of blood smears, which presents the current standard *ante mortem* diagnostic tool [3,59], served to confirm BIBD diagnosis. We prepared two blood smears for each animal, stained with May-Grünwald-Giemsa, and used light microscopy for IB detection in blood cells as described [1]. From the remaining blood, ca. 1 ml each, we separated plasma by centrifugation at 1,200 x g for 2 min, and stored the cell-enriched blood and plasma at -80°C.

### Next generation sequencing (NGS)

NGS served to identify the “reptarenavirome” of the breeding collection, and to allow the setting up of virus-specific RT-PCRs for screening of the entire collection. For NGS, we prepared two pooled samples of cell-enriched blood: 1. three snakes without evidence of BIBD (no IBs in blood cells), 2. three snakes with confirmed BIBD (abundant IBs in blood cells), and performed RNA extraction, NGS library preparation, and genome assembly as described [1,60].

### Reverse transcriptase-polymerase chain reaction (RT-PCR)

We were interested in sequencing the S segments present in the breeding colony, since the S segment bears the NP which we used as the antigen in the antibody assays. As we only recovered a single complete reptarenavirus S segment (University of Giessen virus-1, UGV-1, GenBank accession MH483061) by NGS and *de novo* assembly [10], we decided to use the virus-specific primers of our previous study [1] to screen three additional RNA pools prepared from blood samples by RT-PCR: one BIBD-negative (no evidence of IB in blood cells) and two BIBD-positive. By this approach, we detected: University of Giessen virus-like (UGV-2 and UGV-3, primers [1]), S5-like (S5-like, primers [1]), and Tavallinen suomalainen mies virus-2 (TSMV-2, primers [1]) S segments in the BIBD-positive RNA pools; and S5-like and TSMV-2 S segments in the BIBD-negative RNA pool. We then used these three primer pairs to screen blood samples of the entire collection by RT-PCR. Additionally, we screened the collection by RT-PCR with primers targeting the L segments of two hartmaniviruses identified by NGS and *de novo* assembly in the BIBD positive pool, i.e. Old Schoolhouse viruses 1 and 2 (OScV-1, OScV-2) described in a previous study [10].

We did RNA extractions from cell-enriched EDTA blood (100 μl) as described [1], but introduced a mechanical homogenization step using a Retsch MM300 TissueLyser (QIAGEN) for 2 min at highest frequency (30 Hertz). The following primers were used: UGV-2 and -3 S segment (Fwd 5’-ATAAGGTCAGGGTATAACTTGG-3’ and Rev 5’-GAACTTGGCATAAAAATACAAATGAATG-3’), S5-like S segment (Fwd 5’-GTCAGGATAGAGTCTGGGAGCAT-3’ and Rev 5’-TGAACATTCAGAGGGAATTTGGCATC-3’), TSMV-2 S-segment (Fwd 5’-CAAGTCTGGATAAAGTCTTGGTGCAT-3’ and Rev 5’-GTAATTGATGACGACAATAGGGTCGA-3’), OScV-1 L segment (Fwd 5´- GCACTAAGTGGATCATCAAC-3´ and Rev 5´- CATGCAAACCTGTTGCTG-3´), and OScV-2 L segment (Fwd 5´- GCACTAAGTGGATCATCAAC-3´ and Rev 5´-GAACAATGTCATAACTTGCTC-3´); RT-PCR was performed as described [1], the amplicons analysed by agarose gel electrophoresis, and the bands visualised by GelRed Nucleic Acid Gel Stain (BIOTIUM) under UV-light with the UVP BioDoc-It Imaging System (Thermo Fisher Scientific). The GeneRuler 100 bp DNA ladder (Thermo Fisher Scientific) served as the marker.

### Western blot (WB)

We used UGV-1 virions concentrated by ultracentrifugation through a sucrose cushion, prepared as described in [7], as the antigen in WB. We did the WBs with plasma samples as described in [14], but blocked the nitrocellulose membranes for 3–4 h instead of 30 min at room temperature. We used snake plasma at 1:200 dilution, and the affinity purified unlabelled anti-IgM and anti-IgY antibodies [14] at respective dilutions of 1:500 and 1:1000. We evaluated the results recorded using the Odyssey CLx Infrared Imaging System (LI-COR Biosciences) as negative (–), weakly positive (+), moderately positive (++), and strongly positive (+++) according to the signal intensity.

### Enzyme-linked immunosorbent assay (ELISA)

We set up an ELISA to measure the IgM and IgY levels in the plasma samples using concentrated UGV-1 virions (inactivated with 1% Triton X-100 [Fluka BioChemika]), and recombinant UHV-1 NP and UHV-1 NP-C (described in [61]) as the antigens. We diluted the antigens (UGV-1 at 1:400, UHV-1 NP and UHV-1 NP-C at 2 μg/ml) in 0.05M carbonate buffer, pH 9.6, and used 100 μl/well to coat Nunc Microplate Immuno Polysorp (Thermo Scientific) plates by overnight incubation on an orbital shaker at 4°C. After coating, we used 1% BSA in PBS (150 μl/well) for blocking (2 h at 37°C), washed once with TBS-T (TBS + 0.05% Tween-20) prior to incubation (1 h at 37°C) with the plasma samples diluted (1:200 used for UHV-1 NP-C, and 1:400 for UHV-1 NP and UGV-1) in 0.25% BSA/PBS. After four TBS-T washes, we incubated (45 min at 37°C) the plates with 100 μl/well of horseradish peroxidase (HRP) labelled anti-boa IgM or anti-boa IgY antibodies, described in [14], diluted 1:2000 in 0.25% BSA/PBS, washed four times with TBS-T, incubated (20 min at RT) with TMB Substrate Solution (Thermo Scientific) 100 μl/well, terminated the reaction by addition of 1M H2SO4 50 μl/well, and read the results (OD at 450 nm) with a BioTek Synergy HT Multi-Mode Microplate Reader.

We performed change point analysis utilising the changepoint v.2.2.2 package (https://rdrr.io/cran/changepoint/) in R to set the cut-off values (separately for IgM and IgY and for each antigen) for distinguishing positive and negative ELISA results. Briefly, we used the cpt.meanvar function with the AMOC method on the ELISA data arranged in ascending order. We set the cut-offs (UHV NP IgY = 0.31; UHV NP IgM = 0.35; UGV-1 IgY = 0.27; UGV-1 IgM = 0.48; UHV NP-C IgY = 0.47; and UHV NP-C IgM = 0.37) just above the detected change point, so that the value at change point was considered negative.

### Statistical analysis

We performed data analysis using Stata Statistical Software: Release 13. College Station, TX: StataCorp LP. The analysis examined possible associations between test results and population parameters using univariate and multivariable analysis. For data that were not normally distributed, we utilised non-parametric tests. Given the nature of the investigation and the study population, the analysis is predominantly descriptive. Sensitivity and specificity calculations for the different tests were used as indicative since the study was not designed for the purpose. Cohen’s kappa (κ) and weighted kappa κ (w) served to examine the agreement between tests with binary or ordinal data [42].
